# Volatile Terpenes and Terpenoids from Workers and Queens of *Monomorium chinense* (Hymenoptera: Formicidae)

**DOI:** 10.3390/molecules23112838

**Published:** 2018-11-01

**Authors:** Rui Zhao, Lihua Lu, Qingxing Shi, Jian Chen, Yurong He

**Affiliations:** 1 Department of Entomology, College of Agriculture, South China Agricultural University, Tianhe District, Guangzhou 510642, China; 13570453805@163.com; 2 Plant Protection Research Institute, Guangdong Academy of Agricultural Sciences, Tianhe District, Guangzhou 510640, China; lhlu@gdppri.com (L.L.); shiqingxing163@163.com (Q.S.); 3 National Biological Control Laboratory, Southeast Area, Agriculture Research Service, United States Department of Agriculture, 59 Lee Road, Stoneville, MS 38776, USA

**Keywords:** terpenes, terpenoids, headspace solid phase microextraction, glandular source, *Monomorium chinense*

## Abstract

Twenty-one volatile terpenes and terpenoids were found in *Monomorium* *chinense* Santschi (Hymenoptera: Formicidae), a native Chinese ant, by using headspace solid-phase microextraction (HS-SPME) coupled with gas-phase chromatography and mass spectrometry (GC-MS), which makes this ant one of the most prolific terpene producers in insect. A sesquiterpene with unknown structure (terpene 1) was the main terpene in workers and neocembrene in queens. Terpenes and terpenoids were detected in poison, Dufour’s and mandibular glands of both workers and queens. Worker ants raised on a terpene-free diet showed the same terpene profile as ants collected in the field, indicating that *de* *novo* terpene and terpenoid synthesis occurs in *M*. *chinense*.

## 1. Introduction

Terpenes and terpenoids are the largest group of natural products, mostly produced by plants, but also identified in other eukaryotes such as fungi, insects, amoebae, marine organisms and even prokaryotes, such as, bacteria [1,2,3]. They have drawn great attention from academia and industry due to not only their economic importance in pharmacy, agriculture, food and perfumery industry, but also their ecological significance in mediating antagonistic and beneficial interactions among organisms [4,5,6].

Approximately 55,000 terpenes have been reported in nature [7]. According to our literature survey, a total of 220 terpenes and terpenoids were reported in 9 orders of insects (Blattodea, Coleoptera, Diptera, Heteroptera, Homoptera, Hymenoptera, Isoptera, Lepidoptera and Phasmatodea, Table S1). Among them, about forty-five terpenes or terpenoids originated from ants (Hymenoptera: Formicidae) (Table S1). Terpene and terpenoids play significant roles as pheromones and defense compounds. In the subfamily Formicinae, a wide variety of monoterpenes are utilized as alarm pheromones, such as citronellal, citronellol, α-pinene, β-pinene, limonene and camphene [8]. In genera of *Solenopsis* and *Monomorium* of the subfamily Myrmicinae, farnesenes are often used as trail pheromones [9,10]. In the subfamily Ectatomminae, isogeraniol, a monoterpene might function as a recruitment signal in ant *Rhytidoponera metallica* [11]. In addition to the pheromonal role, terpenes and terpenoids are used as defensive compounds, such as iridomyrmecin (cyclopentanoid monoterpenes) and iridodials in some ant species in the subfamily Dolichoderinae [12,13]. However, functions of many terpenes have not been elucidated, such as (*E*)-β-ocimene and geranylgeraniol in *Labidus praedator* of subfamily Ecitoninae and *Aenictus rotundatus* of subfamily Dorylinae, respectively [14,15].

Terpene and terpenoid biosynthesis have been well studied in plants and microorganisms due to their commercial applications [4]. Terpene biosynthesis and sequestration have also been studied in insects [16,17]. Most insects are herbivores, and previous research showed that terpene sequestration from host plants is common. For example, iridoid glycosides and grayanoid diterpenes were sequestered by certain lepidopteran insects in *Arichanna* and *Euphydryas* from their host plants *Chelone glabra* (Scrophulariaceae) and *Plantago lanceolata* (Plantaginaceae) [18] and the precursor for aggregation pheromone, (−)-*trans*-verbenol, by pine beetles, *Dendroctonus ponderosae*, from pine trees [19]. However, in some herbivorous insect species, terpenes and terpenoids can be produced *de novo,* such as bark beetles and flea beetles, but their biosynthesis pathway of terpenes diverges from that in plants [20,21]. So far, the biosynthesis of terpenes and terpenoids in omnivorous insects like ants has rarely been studied.

*Monomorium chinense* (Hymenoptera: Formicidae) is one of the most dominant ants in the ground ant community, distributed in Palaearctic and Oriental region, and China is the type locality [22]. Although its workers are tiny and look non-aggressive, the ant can succeed in the competition with the notorious invasive ant *Solenopsis invicta* [23]. It is assumed that the exocrine secretions may play a crucial role in the success of *M. chinense*; however, the chemistry of the exocrine glands of this ant species has not been studied. A preliminary study showed that the ant produces an extraordinary number of terpenes and terpenoids. The objective of this study was to identify these terpenes and terpenoids, determine their glandular origins and investigate the effect of diet on terpene composition in order to find out whether *de novo* terpene and terpenoid synthesis occurs in this species of ant.

## 2. Results

### 2.1. Identification of Terpenes and Terpenoids from Whole Bodies of Ants 

**Figure 1:**
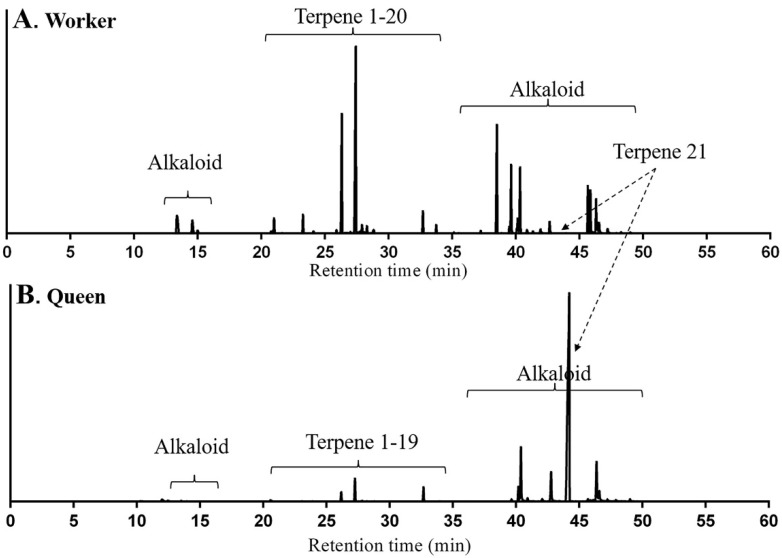
Total ion chromatogram of volatile compounds from *Monomorium chinense* workers (**A**) and queens (**B**) using solid-phase microextraction-gas chromatography-mass spectrometry (SPME-GC-MS) analysis with a DB-5 capillary column.

Total ion chromatograms (TICs) of volatile compounds extracted by solid-phase microextraction (SPME) from *M. chinense* workers and queens are shown in Figure 1. In addition to alkaloids, terpenes and terpenoids were major volatile compounds which are listed in Table 1. For peaks 1, 5 to 9, 11 to 12, 14 to 16 and 18 to 19, compounds were identified as δ-elemene, β-acoradiene, α-neocallitropsene, β-chamigrene, γ-curcumene, aristolochene, β-himachalene, (*Z*)-α-bisabolene, β-curcumene, 7-epi-α-selinene, β-sesquiphellandrene, γ-cuprenene and 8-cedren-13-ol respectively by comparing their retention times (RTs), Arithmetic indexes (AIs), Kovǎts indexes (KIs) and mass spectra with compounds in the literatures (Figures S1, S5–S9, S11, S12, S14–S16, S18 and S19). For peak 2, 3 and 4, compounds were identified and confirmed as β-elemene, β-cedrene and (*E*)-β-farnesene respectively using authentic standards (Figures S2–S4). For peak 21, the compound was identified as neocembrene, a diterpene, since it had RT, AI, KI and mass spectrum matched with neocembrene gas chromatography and mass spectrometry (GC-MS) peak in *M. pharaonis* (Figure S21) [24]. 

The identities of peaks 10, 13, 17, and 20 could not be finalized because there was no match in RTs with available standards, no match in KIs and AIs with any terpenes and terpenoids in the literature. Therefore the mass spectra of those peaks are presented: peak 10 (terpene 1), [55(52), 79(88), 93(84), 105(92), 119(55), 133(87), 161(100), 175(51), 189(67), 204(78)]; peak 13 (terpene 2), [55(17), 79(22), 93(25), 105(70), 119(54), 133(35), 148(13), 161(97), 189(82), 204(100)]; peak 17 (terpene 3) [55(22), 79(28) 93(40), 105(93), 119(69), 133(44), 148(13), 161(100), 189(43), 204(77)]; and peak 20 (terpenoid 1) [55(49), 67(57), 81(69), 93(71), 107(100), 121(67), 147(34), 161(33), 175(40), 189(58), 217(38), 232(50)]. Peaks 10, 13, 17 are sequiterpenes with molecular ions at *m*/*z* 204, predicting their molecular formula C_15_H_24_. The mass spectrum of terpene 1 is similar to that of (5R*,7R*,10S*)-selina-4(14), 11-diene found in *Nasutitermes* [25]. Peak 20 (terpenoids 1) was considered sesquiterpenoid based on its molecular ion at *m*/*z* 232, predicting its molecular formulas C_15_H_20_O_2_. Mass spectra of other unknown terpenes from the ant were provided as well in the supporting material (Figures S10, S13, S17 and S20).

The squared Mahalanobis distance of terpene composition between workers and queens was 53.22 (*F* = 29.94, *p* < 0.05), indicating the significant difference between two groups. The relative content of terpene 1 in workers was 48.15 ± 2.97% of all compounds as a dominant terpene, followed by β-acoradiene (24.13 ± 2.11%). The other 19 terpenes and terpenoids in a small quantity accounted for 27.72% in average. Terpenoid 1 was only found in workers, but not in queens. For queens, neocembrene accounted for 89.00 ± 1.46% of all compounds as a dominant terpene, but the other 19 terpenes and terpenoids in a small amount accounted for 11.00% in average. 

### 2.2. Origin of Terpenes and Terpenoids

#### 2.2.1. Body Parts

**Figure 2:**
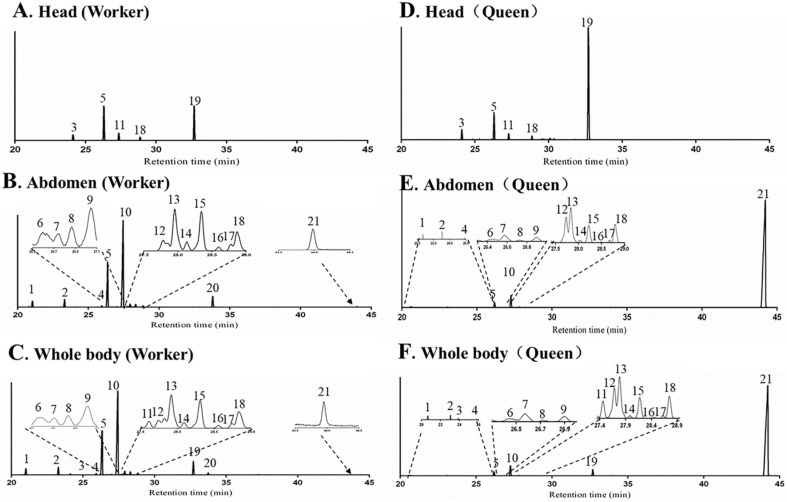
Total ion chromatograms (TICs) of volatile terpenes and terpenoids from the head (**A**), abdomen (**B**) and whole body (**C**) extracts in workers, head (**D**), abdomen (**E**) and whole body (**F**) extracts in queens of *Monomorium chinense* using SPME-GC-MS analysis with a DB-5 capillary column.

Twenty terpenes and terpenoids were found from whole body samples of both workers and queens, including nineteen sesquiterpenes and sesquiterpenoids (peak 1–19) and one diterpene (peak 21). One sesquiterpenoid, terpenoid 1 (peak 20) appeared only in the workers (Figure 2). TICs of different body parts revealed that abdomen and head were the major sources of terpenes, however no terpene or terpenoid was found in the thorax. For workers and queens, β-acoradiene (Peak 5) and γ-cuprenene (peak 18) were detected in both head and abdomen; and δ-elemene (peak 1), β-elemene (peak 2), (*E*)-β-farnesene (peak 4), α-neocallitropsene (peak 6), β-chamigrene (peak 7), γ-curcumene (peak 8), aristolochene (peak 9), terpene 1 (peak 10), (*Z*)-α-bisabolene (peak 12), terpene 2 (peak 13), β-curcumene (peak 14), 7-epi-α-selinene (peak 15), β-sesquiphellandrene (peak 16), terpene 3 (peak 17) and neocembrene (peak 21) only in the abdomen; β-cedrene (peak 3), β-himachalene (peak 11) and 8-cedren-13-ol (peak 19) only in the head. Terpenoid 1 (peak 20) was only found in the abdomen of workers.

#### 2.2.2. Exocrine Glands

**Figure 3:**
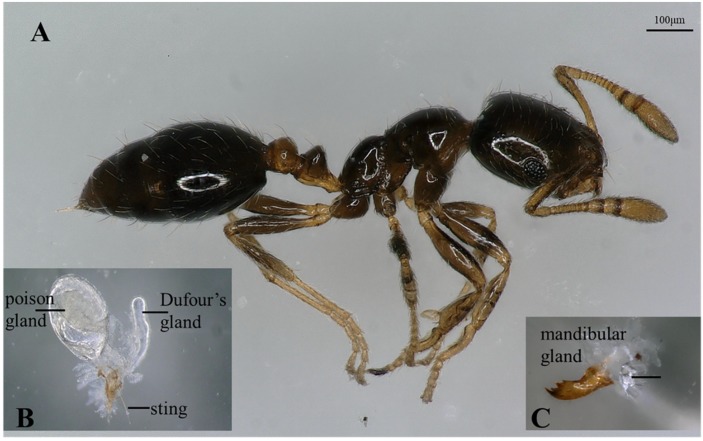
Lateral view of a worker (**A**), poison, Dufour’s (**B**), and mandibular glands (**C**) of *Monomorium chinense* workers.

**Figure 4:**
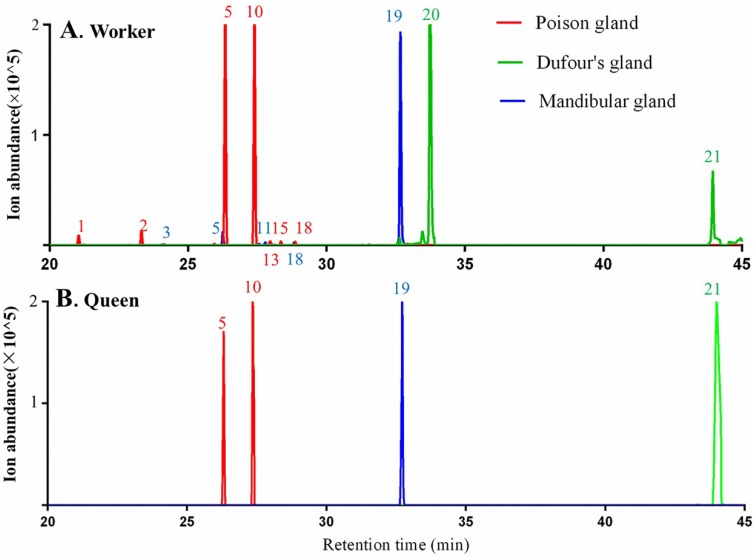
TICs of terpenes and terpenoids from the poison glands, Dufour’s glands and mandibular glands of *Monomorium chinense* workers (**A**) and queens (**B**) using SPME-GC-MS analysis with a DB-5 capillary column.

In the workers of *M. chinense*, poison and Dufour’s glands in the abdomen and mandibular gland in the head were dissected (Figure 3). Seven terpenes including δ-elemene (peak 1), β-elemene (peak 2), β-acoradiene (peak 5), terpene 1 (peak 10), terpene 2 (peak 13), 7-epi-α-selinene (peak 15) and γ-cuprenene (peak 18) were detected in the poison gland, terpenoid 1 (peak 20) and neocembrene (peak 21) in the Dufour’s gland, and five terpenes and terpenoids including β-cedrene (peak 3), β-acoradiene (peak 5), β-himachalene (peak 11), γ-cuprenene (peak 18) and 8-cedren-13-ol (peak 19), in the mandibular gland (Figure 4A). 

In queens, β-acoradiene (peak 5) and terpene 1 (peak 10) were detected in the poison gland, neocembrene (peak 21) in the Dufour’s gland, and 8-cedren-13-ol (peak 19) in the mandibular gland (Figure 4B).

Some terpenes and terpenoids found in ant body parts were not detected in gland samples. For example, nine terpenes in the abdomen of workers and queens were not detected in both poison and Dufour’s glands, including α-neocallitropsene (peak 6), β-chamigrene (peak 7), γ-curcumene (peak 8), aristolochene (peak 9), (*Z*)-α-bisabolene (peak 12), terpene 2 (peak 13), β-curcumene (peak 14), β-sesquiphellandrene (peak 16) and terpene 3 (peak 17). Reduced abundance of all these compounds due to their evaporation during the dissection process may be the reason why they could not be detected by GC-MS in gland samples. 

#### 2.2.3. Influence of Diet on Terpene and Terpenoid Profile 

**Figure 5:**
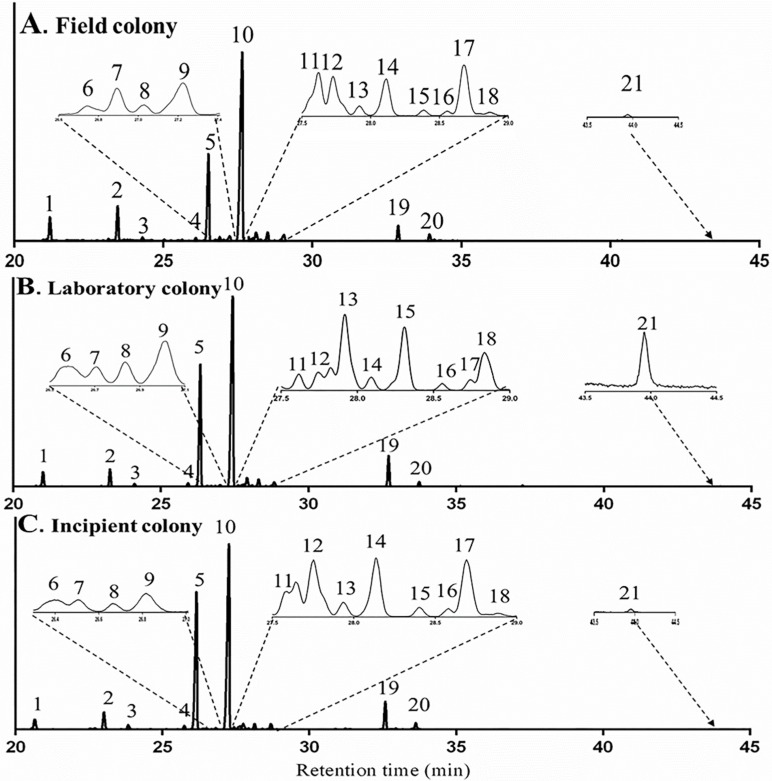
TICs of terpenes and terpenoids of *Monomorium chinense* workers from a field colony (**A**), a laboratory colony (**B**) and an incipient colony (**C**).

TICs of whole-body samples of workers for a field colony (unknown diet), a laboratory colony (controlled diet: mealworm larvae and honey water) and an incipient colonies (terpene-free diet: sucrose water) are shown in Figure 5. Twenty-one terpenes and terpenoids were detected in both the field colonies and the laboratory colonies, in which natural diet were provided, all these compounds were observed also in the incipient colonies, which were fed with the sucrose solution. The squared Mahalanobis distance between field colonies and laboratory colonies, field colonies and incipient colonies, laboratory colonies and incipient colonies was 10.95 (*F* = 3.65, *p* = 0.12), 12.86 (*F* = 4.29, *p* = 0.09) and 7.88 (*F* = 2.63, *p* = 0.18), respectively and all *p* values were above 0.05, indicating that there was no significant difference of terpene contents among three treatments. Therefore, the terpenes and terpenoids, found in *M. chinense* workers in different treatments were not sequestered from their dietary sources.

## 3. Discussion

Twenty-one terpenes and terpenoids were detected from workers and twenty from queens of *M. chinense*. Previous studies showed that *Pheidole sinaitica* and *Solenopsis geminata* are the top terpene producers in ants (Hymenoptera: Formicidae) in term of numbers of terpenes and terpenoids detected. For example, the minor workers of *P*. *sinaitica* contained a mixture of more than 11 sesquiterpenes (farnesene-type hydrocarbons) [26]. The *S*. *geminata* queens produced 11 sesquiterpenes in the venom secretion and among them β-elemene was only tentatively identified [27]. The results indicate that *M. chinense* is an exceptional terpene producing ant. 

In insects, 60 terpenes and terpenoids have been discovered from papilionid larvae (Lepidoptera: Papilionidae) and 53 from termite soldiers (Isoptera: Rhinotermitidae) (Table S1). They seem to be the top terpene and terpenoid producers in insects. In addition to 21 terpenes and terpenoids identified in *M*. *chinense*, 10 terpenes and terpenoids have been reported in *M*. *minimum* and five in *M*. *pharaonic* [28]. These results suggest that *Monomorium* ants may be one of the most potent terpene producers in insects. 

To the best of our knowledge, α-neocallitropsene, β-chamigrene and 8-cedren-13-ol have never been reported in insects. The following 10 sesquiterpenes were found for the first time in ants, including δ-elemene, β-cedrene, γ-curcumene, aristolochene, β-himachalene, (*Z*)-α-bisabolene, β-curcumene, β-sesquiphellandrene, γ-cuprenene, and 7-epi-α-selinene. Some terpenes found in this study have already been reported in other insects. For example, β-elemene was detected in termite *Reticulitermes speratus* (Isoptera: Rhinotermitidae), δ-elemene, γ-curcumene, β-cedrene, β-himachalene, and β-acoradiene in butterflies (Lepidoptera: Papilionidae), β-sesquiphellandrene in stinkbugs *Thyanta pallidovirens* and *Piezodorus guildinii* (Hemiptera: Pentatomidae), and β-curcumene in ciid beetles *Octotemnus glabriculus* and *Cis boleti* (Coleoptera: Ciidae). Terpenes play multiple functions in these insects, serving as inhibitory primer pheromones, queen-recognition and sex pheromones, defensive chemicals against natural enemies, and antimicrobials against pathogens [29,30,31,32,33,34]. Further research is needed to determine whether terpenes and terpenoids play similar roles in *M. chinense.*

Terpene 1, a sesquiterpene with unknown structure, was the main terpene in workers, followed by β-acoradiene and the remaining terpenes and terpenoids are all minor products. In addition to the major product, nearly half of all characterized monoterpene and sesquiterpene synthases in plants form significant number of minor products [35]. For example, the major sesquiterpene product of valencene synthase was identified as (+)-valencene (49.5% of total product), followed by (−)-7-epi-α-selinene (35.5%) along with five minor products [36]. It is possible that one or few terpene synthases in the workers of *M*. *chinense* may be responsible for such a diversity of terpenes and terpenoids. 

Usually only one type of gland is involved in terpene and terpenoid production and/or storage in one species of insects, such as osmeteria glands in Papilionid (Lepidoptera: Papilionidae) larvae, frontal glands in termite soldiers (Isoptera: Rhinotermitidae, Serritermitidae, and Termitidae), and metasternal glands in longhorned beetles (Coleoptera: Cerambycidae) [37,38,39]. In contrast, in this study, the terpenes and terpenoids have been detected in three glands, including poison, Dufour’s and mandibular glands in *M. chinense* workers and queens. A list of terpenes or terpenoids in ants with glandular source is summarized in Table S2. Monoterpenes and monoterpenoids have been discovered in rectum, mandibular, Dufour’s, poison and pygidial glands in Formicinae, Myrmicinae, Dorylinae and Dolichoderinae. Although sesquiterpenes and diterpenes were mostly found from Dufour’s gland, they were also detected in Mandibular, Dufour’s or venom glands in Formicinae, Myrmicinae and Nothomyrmeciinae. The multiglandular origin of terpenes and terpenoids may make *M. chinense* a unique case in family Fomicidae, maybe even in the class Insecta.

Neocembrene was the major terpene produced in the Dufour’s gland of *M. chinense* queens in contrast to its minor abundance in workers. This compound was found in the Dufour’s gland in *M. pharaonis* queens, but not in the workers [24]. Whether neocembrene serves as queen pheromone in *M. pharaonis* remains questionable because it does not affect sexual brood rearing [40]. Besides two ant species mentioned above, neocembrene was detected in queens of other four species in the genus *Monomorium*, including *M. minimum* [41], *M. floricola*, *M. destructor* and *M. hiten* [42], indicating that neocembrene may be a genus- and queen-specific compound in genus *Monomorium.* Terpenes and terpenoids do not occur often in poison gland. When limonene, a monoterpene, was first found in poison glands of *Myrmicaria* species, it was considered an unusual case in Formicidae [43]. This study reports that sequiterpenes occur in the poison glands of worker ants. Typical poison gland chemistry of *Monomorium* species was dominated by alkaloids, which were believed to be the reason for them to successfully compete with the highly aggressive ant species [44,45]. This study reveals that not only alkaloids but also sesquiterpenes occur in the poison glands of *M. chinense* workers. Along with alkaloids, terpenes from the poison gland may act synergistically to provide higher toxicity or deterrence. However, the specific functions of these terpenes and terpenoids can only be clarified in the future research.

In plants, terpenoids function universally as primary metabolites, such as sterols, carotenoids, quinones, and hormones [46]. However, most of terpenes and terpenoids in plants are restricted to specific lineages and are involved in species-specific ecological interactions as secondary metabolites that may serve roles in plant defense and communication [47]. Terpenes identified in *M. chinense*, *M. pharaonis* and *M. minimum*, do not occur in genera outside *Monomorium*, indicating these terpenes and terpenoids may also lineage-specific (specialized) terpenoids. Thus, they are most likely also involved in the interaction with other organisms and environment, such as defense against enemies and diseases, or conspecific and heterospecific chemical communications.

All terpenes and terpenoids identified in this study have been found in plants. *M. chinense* is an omnivorous ant as other species in the genus *Monomorium* [48], so it was hypothesized that their diet is one potential source of these terpenes and terpenoids. However, ants raised on a terpene-free diet showed the same terpene profile as those of ants fed with natural diets, indicating that *de novo* terpene synthesis occurs in *M. chinense*. The terpene biosynthesis of bark beetles and flea beetles is well studied. Both beetles are oligophagous herbivores. Ivarsson et al. provided the first evidence that bark beetle *Ips duplicatus* can produce their main pheromone component, ipsdienol, a terpene alcohol [49]. Radiolabeling studies provided further evidence of the *de novo* biosynthesis of terpenes by bark beetles [50]. Geranyl diphosphate synthase of bark beetle *Ips pini* is the first animal prenyltransferase having terpene synthase activity [21]. No sesquiterpene synthases have been described in insects until the identification of an evolutionarily novel terpene synthase gene family in the striped flea beetle [51]. Terpene and terpenoid biosynthesis in ants have not really been studied by researchers. Considering the significance of terpenes and terpenoids in pharmacy, agriculture, food and perfumery industry, understanding and characterizing terpene synthases in ants may become important, since ant terpene synthase genes may provide us with new opportunities in bioengineering for production of high-valued terpenes and terpenoids. Due to its exceptional ability in terpene production, *M. chinense* may be a good model insect for study terpene biosynthesis in ants.

## 4. Materials and Methods

### 4.1. Ants

#### 4.1.1. Maintenance of Field-Collected Ant Colonies 

Nine colonies of *M. chinense* were collected in Guangzhou, Guangdong, China, and among them 3 colonies were collected from the campus of Guangdong Academy of Agriculture Science (GAAS) in July 2016, 3 from Baiyun district in April 2017 and 3 from Nansha district in April 2017. The colonies were reared in a 45 × 38 × 15 cm plastic container with the inner sides of the wall coated with Fluon F4-1 (Xingshengjie Sci and Tech Co., Ltd., Guangzhou, China) to prevent the escape of ants. Three glass test tubes (2.5 Φ × 19.5 cm) were placed in the container and used as artificial nests. Each tube was filled with 4–5 cm of water and a cotton plug was placed in the tube at the water level to retain the water. Tubes were covered with black paper to shield the light. Colonies were provided with minced mealworm, *Tenebrio molitor*, a cotton ball saturated with a 20% honey water solution, and a cotton ball with pure water in a Petri dish (7 × 1.5 cm). These colonies were maintained at 26 ± 2 °C and 12:12 (L:D) h photoperiod.

#### 4.1.2. Establishment of Incipient Colonies 

Incipient colonies were established by introducing newly dealate queens with 20 workers from the laboratory colonies into a container (45 × 38 × 15 cm). Once young workers emerged in the new colony, the old workers were removed. Colonies were provided with a cotton ball saturated with a 20% sucrose water solution, and a cotton ball with pure water in a Petri dish (7 × 1.5 m). They were maintained at 26 ± 2 °C and 12:12 (L:D) h photoperiod.

### 4.2. Chemical Analysis of Ant Volatile Terpenes and Terpenoids

#### 4.2.1. Ant Sample Preparation and Extraction by HS-SPME 

About 100 live ant workers or 10 queens were put into a 2 mL vial (Agilent Technologies, Santa Clara, CA, USA). In order to facilitate the release of volatiles from the sample into the head space, the vial was placed into a −80 °C refrigerator for 10 min [28]. Headspace solid-phase micro-extraction (HS-SPME) was then conducted on the sample at room temperature (25 ± 1 °C) for 12 h using an 85 μm Polyacrylate SPME fiber (Supelco Inc., Bellefonte, PA, USA). In order to add C_8_ to C_20_ hydrocarbon standards to the sample, after the sample extraction, the same fiber was used to extract hydrocarbon standards for 1 min in another 2 mL vial. The hydrocarbon standards were prepared by adding 20 µL C_8_ to C_20_ solution (Sigma-Aldrich, St. Louis, MO, USA, 40 mg/L) into the vial and letting solvent evaporate in a fume hood. In order to facilitate evaporation of the solvent, the capped vial was shaken for 5 s before it was opened in a fume hood. After 1 min of evaporation, the vial was capped and shaken again for 5 s before it was reopened in the fume hood for 9 min. Before each SPME sample extraction, a blank run was performed and the fiber was cleaned in the GC injector for 30 min. There were 5 replicates for each colony.

#### 4.2.2. Determination of Glandular Sources of Terpenes and Terpenoids 

Each worker or queen was cut into three major body parts (head, thorax and abdomen) by a razor blade. Each type of body parts was placed into 2 mL vial that was subjected to SPME extraction as described as above. Since terpenes and terpenoids were found in the head and abdomen, the chemistry of the poison gland and Dufour’s gland in abdomen and mandibular gland in head were investigated. Because poison gland and Dufour’s gland are connected, they were first removed from the body under a stereo microscope (SZ61, Olympus, Tokyo, Japan) by grasping the terminal abdominal segments or the stinger with fine forceps and pulling posteriorly. The poison and Dufour’s glands were separated with a dissecting needle. The mandibular gland was removed by grasping the mandible away from head, then separating the gland using a dissecting needle. After separation, each gland was directly placed on the tip of the SPME fiber, which then was inserted into the inject port of the GC-MS system (Agilent Technologies, Santa Clara, CA, USA). 

#### 4.2.3. Gas Chromatography and Mass Spectrometry (GC-MS) 

The samples were analyzed using GC-MS Agilent 7890A-gas chromatograph coupled with 5975B-mass spectrometer. The analytical conditions were used as follows, splitless injection at 250 °C, DB-5 column (30 m × 0.25 mm i.d., 0.25 μm film thickness), the temperature program was from 60 °C to 246 °C at 3 °C. min^−1^. Injector temperature was 220 °C and transfer line temperature 240 °C. The mass spectrometer was operated at 70 eV in the electron impact mode.

### 4.3. Data Analysis 

Arithmetic index (AI) and Kovǎts index (KI) of target compounds were calculated using the following formula [52]:

KI (x) = 100 P_Z_ + 100 [(log RT (x) − log RT (P_Z_))/(log RT (P_Z+1_) − log RT (P_Z_))]

AI (x) = 100 P_Z_ + 100 [(RT (x) − RT (P_Z_))/(RT (P_Z+1_) − RT (P_Z_))]

where: RT (P_Z_) ≤ RT (x) ≤ RT (P_Z+1_), and P_8_…P_20_ were *n*-paraffins. (up to N = 20 in the paper).

Terpenes and terpenoids were identified by comparing retention times (RT), AIs and KIs, and mass spectra of compounds with synthetic standards and compounds in literature [51] and libraries [NIST (National Institute of Standards and Technology, Gaithersburg, MD, USA) and Wiley (John Wiley & Sons, Inc., Hoboken, New Jersey, USA)]. Synthesized compounds of β-elemene, β-cedrene and (*E*)-β-farnesene were purchased from Sigma-Aldrich (St. Louis, MO, USA). Since neocembrene was originally identified in *Monomorium pharaonis* queens [24], the neocembrene extracted from *M*. *pharaonis* queens was used as a standard for identification of the compound in *M*. *chinense*. The pharaoh ant colonies were reared in the Laboratory of Biological Invasion, Plant Protection Research Institute, GAAS.

Relative peak area of each terpene or terpenoid was calculated in percentage over the total area of all peaks. To estimate the difference of terpene composition between worker and queens, and the difference among three groups (field colonies, laboratory colonies and incipient colonies), a total of 21 terpene peak relative contents were used as variables in a principal component analysis and the principal components extracted were used as independent variables in the subsequent discriminant analysis and the squared Mahalanobis distances (D2) between the clusters were calculated. There were 3 replicates for each colony. STATISTICA 10.0 (Palo Alto, CA, USA), was used in statistical analyses.

## 5. Conclusions

In summary, twenty-one volatile terpenes and terpenoids were found in the Chinese ant, *Monomorium chinense* using headspace solid-phase micro-extraction (HS-SPME) coupled with gas-phase chromatography and mass spectrometry (GC-MS). The discovery makes *M. chinense* the most prolific terpene producer in ants. A sesquiterpene with unknown structure terpene 1 and neocembrene are the main terpene in the workers and queens, respectively. Most terpenes and terpenoids were found in the poison, Dufour’s and/or mandibular glands. *De novo* terpenes and terpenoids synthesis are demonstrated in in *M. chinense* its workers. These findings suggest *M. chinense* is a novel and promising organism for the study of terpene function and biosynthesis in ants.

## Figures and Tables

**Table 1:** Volatile terpenes and terpenoids from workers and queens of *Monomorium chinense*.

Peak No.	Compound	RT (min)	AI	KI	Identification Proposal *	Glandular Source **		Relative Content (Mean ± SE) (%)	
						Worker	Queen	Worker	Queen
1	δ-elemene	21.024	1338	1341	B	PG	Abd	2.7 ± 0.44	0.29 ± 0.14
2	β-elemene	23.292	1392	1393	A	PG	Abd	3.11 ± 0.77	0.3 ± 0.12
3	β-cedrene	24.436	1420	1421	A	MG	Head	0.71 ± 0.19	0.07 ± 0.05
4	(*E*)-β-farnesene	25.917	1457	1459	A	PG	Abd	0.76 ± 0.28	0.03 ± 0.01
5	β-acoradiene	26.323	1467	1469	B	MG, PG	PG, Head	24.13 ± 2.11	2.01 ± 0.18
6	α-neocallitropsene	26.569	1473	1475	B	Abd	Abd	0.24 ± 0.09	0.02 ± 0.00
7	β-chamigrene	26.708	1477	1478	B	Abd	Abd	0.7 ± 0.28	0.04 ± 0.01
8	γ-curcumene	26.836	1480	1481	B	Abd	Abd	0.22 ± 0.14	0.01 ± 0.01
9	aristolochene	27.012	1484	1485	B	Abd	Abd	0.62 ± 0.49	0.31 ± 0.05
10	terpene 1	27.4	1494	1494	C	PG	PG	48.15 ± 2.97	4.75 ± 0.48
11	β-himachalene	27.617	1499	1499	B	MG	Head	0.84 ± 0.72	0.14 ± 0.11
12	(*Z*)-α-bisabolene	27.747	1503	1503	B	Abd	Abd	0.63 ± 0.47	0.23 ± 0.12
13	terpene 2	27.974	1509	1509	C	PG	Abd	1.99 ± 0.85	0.15 ± 0.11
14	β-curcumene	28.093	1512	1512	B	Abd	Abd	0.33 ± 0.14	0.07 ± 0.01
15	7-epi-α-Selinene	28.311	1517	1518	B	PG	Abd	1.51 ± 0.2	0.07 ± 0.05
16	β-sesquiphellandrene	28.577	1524	1525	B	Abd	Abd	0.59 ± 0.44	0.05 ± 0.04
17	terpene 3	28.743	1529	1530	C	Abd	Abd	1.19 ± 0.81	0.01 ± 0.00
18	γ-cuprenene	28.853	1531	1533	B	MG, PG	Abd, Head	1.68 ± 0.72	0.09 ± 0.04
19	8-cedren-13-ol	32.721	1633	1634	B	MG	MG	7.04 ± 5.08	2.34 ± 0.62
20	terpenoid 1	33.751	1661	1662	C	DG	-	2.51 ± 0.67	0
21	neocembrene	43.951	1959	1959	B	DG	DG	0.33 ± 0.27	89.00 ± 1.46

* The reliability of the identification proposal is indicated by the following: A, mass spectrum, arithmetic index (AI) and Kovǎts index (KI) agreed with the standards; B, mass spectrum, arithmetic index and Kovǎts index agreed with literature data; C, unidentified terpenes or terpenoids, indicating no match of mass spectrum with standards & literature data & mass spectral database. ** Poison gland (PG); Dufour’s gland (DG); mandibular gland (MG); abdomen (Abd).

## Supplementary Materials

The following are available online. Mass spectra of terpenes and terpenoids showed in figures (Figures S1–S21); Terpenes and terpenoids in insects, terpenes and terpenoids in ants and their glandular source summarized in tables (Tables S1 and S2).
