# Supplementary material for: Volatile Terpenes and Terpenoids from Workers and Queens of Monomorium chinense (Hymenoptera: Formicidae)
Source: Molecules. 2018 Nov 1;23(11):2838. doi: 10.3390/molecules23112838 (PMC6278355; doi:10.3390/molecules23112838)
Supplement: Supplementary file 1 [file molecules-23-02838-s001.pdf]

SUPPLEMENTARY MATERIAL

Volatile Terpenes and Terpenoids from the Workers and Queens of *Monomorium chinense* (Hymenoptera: Formicidae)

Rui Zhao [1, 2], Lihua Lu [2], Qingxing Shi [2], Jian Chen [3*], Yurong He [1*]

[1] Department of Entomology, College of Agriculture, South China Agricultural University, Tianhe District, Guangzhou, Guangdong 510642, China; 13570453805@163.com (R.Z.)

[2] Plant Protection Research Institute, Guangdong Academy of Agricultural Sciences, Tianhe District, Guangzhou, Guangdong 510640, China; lhlu@gdppri.com (L.L.); shiqingxing163@163.com (Q.S.)

[3] National Biological Control Laboratory, Southeast Area, Agriculture Research Service, United States Department of Agriculture, 59 Lee Road, Stoneville, MS 38776, USA

*Correspondence: jian.chen@ars.usda.gov (J.C.); yrhe@scau.edu.cn (Y.H.)

Table of Contents

Figure S1 Mass spectrum of peak 1 (a) from *Monomorium chinense* workers and δ-elemene (b) from the literature ~~book~~, showing the match of mass spectra. 3

Figure S2 Total ion chromatograms of extract of *Monomorium chinense* workers (a) and standard β-elemene (b), showing the match of retention times; Mass spectra of peak 2(c) and standard β-elemene (d), showing the match of mass spectra. 3

Figure S3 Total ion chromatograms of extract of *Monomorium chinense* workers (a) and standard β- cedrene (b), showing the match of retention times; Mass spectra of peak 3(c) and standard β-cedrene (d), showing the match of mass spectra. 4

Figure S4 Total ion chromatograms of extract of *Monomorium chinense* workers (a) and standard (E)-β-farnesene (b), showing the match of retention times; Mass spectra of peak 4(c) and standard (E)-β-farnesene (d), showing the match of mass spectra. 4

Figure S5 Mass spectra of peak 5 (a) from *Monomorium chinense* workers and β-acoradiene (b) from the literature ~~book~~, showing the match of mass spectra. 5

Figure S6 Mass spectra of peak 6 (a) from *Monomorium chinense* workers and α-neocallitropsene (b) from the literature ~~book~~, showing the match of mass spectra. 5

Figure S7 Mass spectra of peak 7 (a) from *Monomorium chinense* workers and β-chamigrene (b) from the literature ~~book~~, showing the match of mass spectra. 6

Figure S8 Mass spectra of peak 8 (a) from *Monomorium chinense* workers and γ-curcumene (b) from the literature ~~book~~, showing the match of mass spectra. 6

Figure S9 Mass spectra of peak 9 (a) from *Monomorium chinense* workers and aristolochene (b) from the literature ~~book~~, showing the match of mass spectra. 7

Figure S10 Mass spectrum of p10 from *Monomorium chinense* workers. 7

Figure S11 Mass spectra of peak 11 (a) from *Monomorium chinense* workers and β-himachalene (b) from the literature ~~book~~, showing the match of mass spectra. 8

Figure S12 Mass spectra of peak 12 (a) from *Monomorium chinense* workers and (Z)-α-bisabolene (b) from the literature ~~book~~, showing the match of mass spectra. 8

Figure S13 Mass spectrum of p13 from *Monomorium chinense* workers. 9

Figure S14 Mass spectra of peak 14 (a) from *Monomorium chinense* workers and β-curcumene (b) from the literature ~~book~~, showing the match of mass spectra. 9

Figure S15 Mass spectra of peak 15 (a) from *Monomorium chinense* workers and 7-epi-α-selinene (b) from the literature ~~book~~, showing the match of mass spectra. 10

Figure S16 Mass spectra of peak 16 (a) from *Monomorium chinense* workers and β-sesquiphellandrene (b) from the literature ~~book~~, showing the match of mass spectra. 10

Figure S17 Mass spectrum of p17 from *Monomorium chinense* workers. 11

Figure S18 Mass spectra of peak 18 (a) from *Monomorium chinense* workers whole body **extractio**n and γ-cuprenene (b) from the literature ~~book~~, showing the match of mass spectra. 11

Figure S19 Mass spectra of peak 19 (a) from *Monomorium chinense* workers and 8-cedren-13-ol (b) from the literature ~~book~~, showing the match of mass spectra. 12

Figure S20 Mass spectrum of p20 from *Monomorium chinense* workers. 12

Figure S21 Total ion chromatograms of extract of *Monomorium chinense* workers (a) and *Monomorium pharaonis* workers (b), showing the match of retention times; Mass spectra of peak 2(c) and neocembrene (d), showing the match of mass spectra. 13

Table S1 Terpenes and terpenoids in insects. 14

Table S2 Terpenes and terpenoids in ants and their glandular source. 15

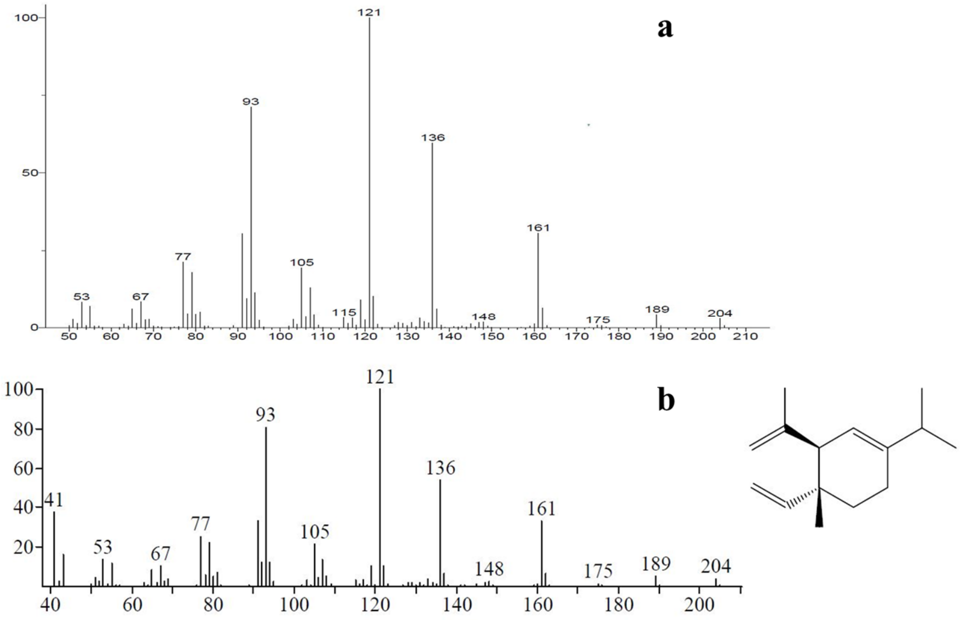

Figure S1 Mass spectrum of peak 1 (a) from *Monomorium chinense* workers and δ-elemene (b) from the literature book, showing the match of mass spectra.

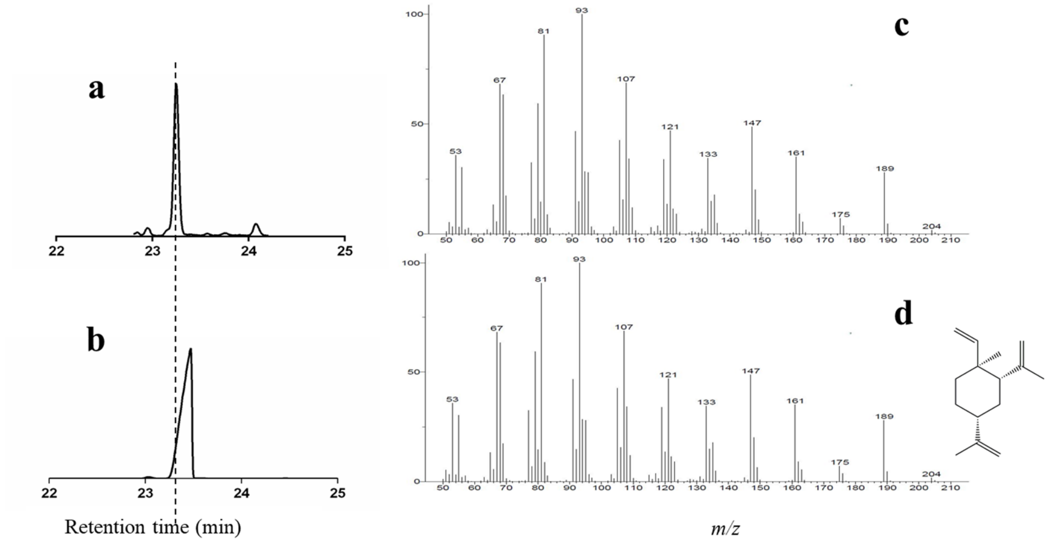

Figure S2 Total ion chromatograms of extract of *Monomorium chinense* workers (a) and standard β-elemene (b), showing the match of retention times; Mass spectra of peak 2(c) and standard β-elemene (d), showing the match of mass spectra.

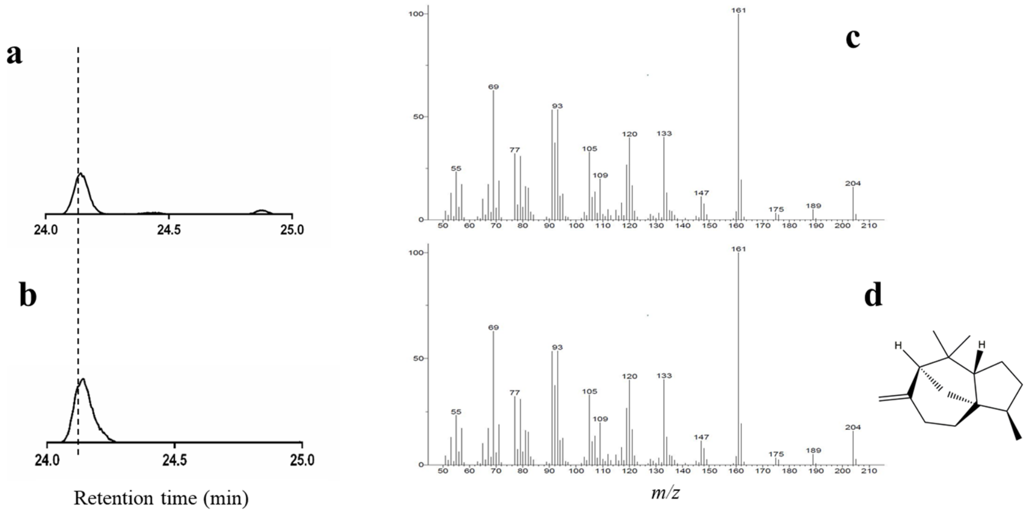

Figure S3 Total ion chromatograms of extract of *Monomorium chinense* workers (a) and standard β- cedrene (b), showing the match of retention times; Mass spectra of peak 3(c) and standard β-cedrene (d), showing the match of mass spectra.

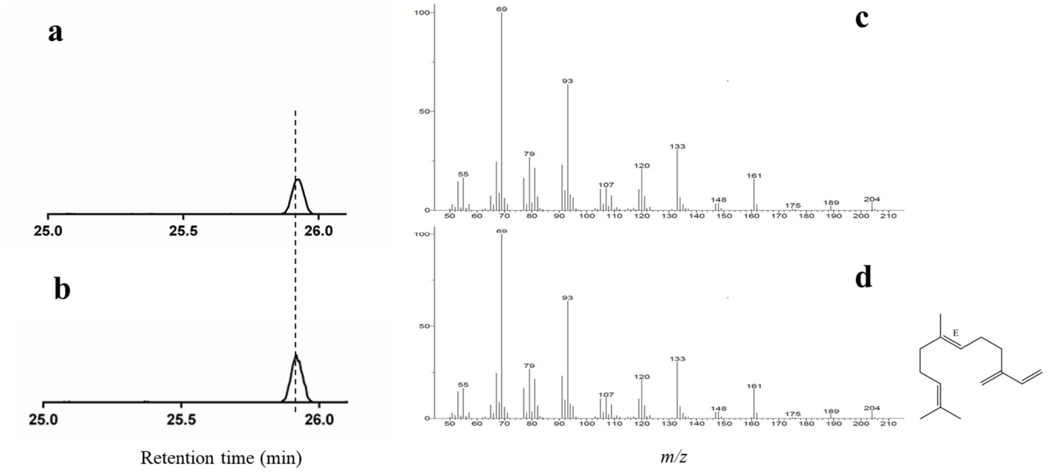

Figure S4 Total ion chromatograms of extract of *Monomorium chinense* workers (a) and standard (E)-β-farnesene (b), showing the match of retention times; Mass spectra of peak 4(c) and standard (E)-β-farnesene (d), showing the match of mass spectra.

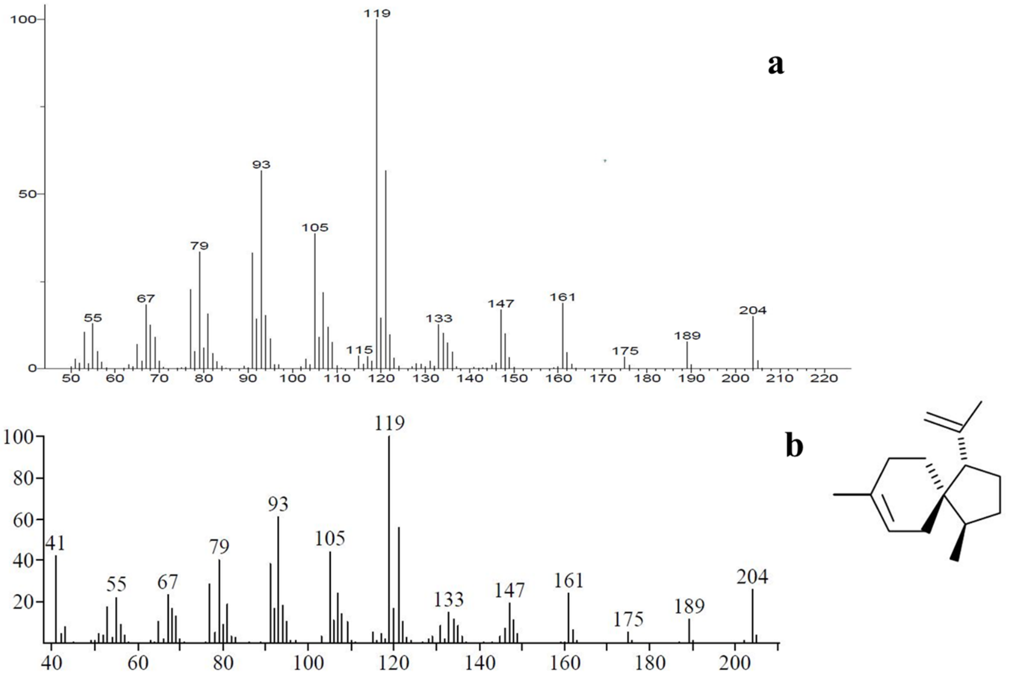

Figure S5 Mass spectra of peak 5 (a) from *Monomorium chinense* workers and β-acoradiene (b) from the literature book, showing the match of mass spectra.

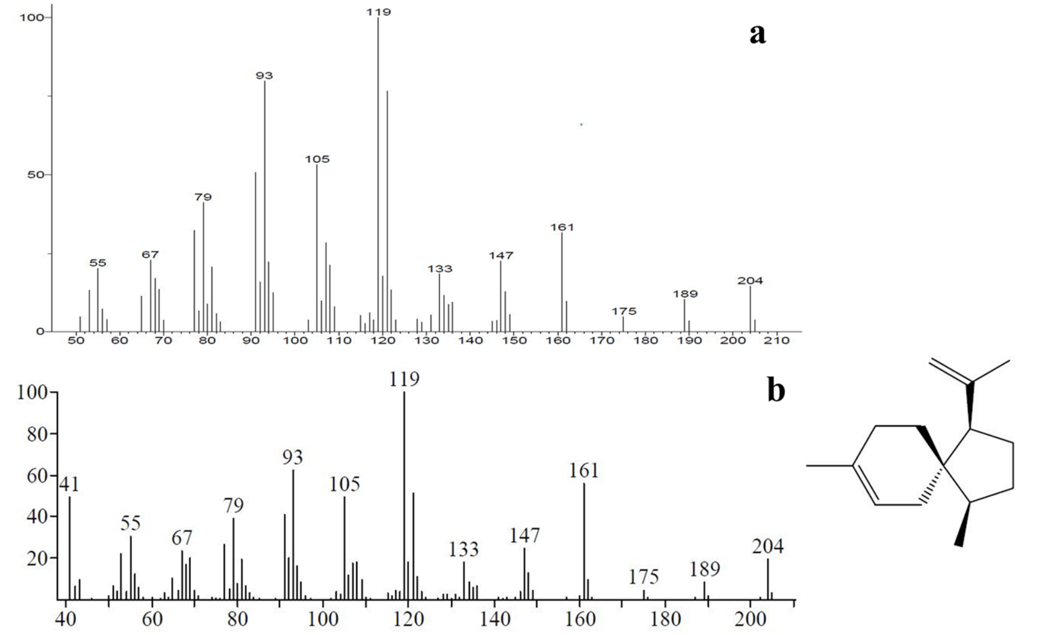

Figure S6 Mass spectra of peak 6 (a) from *Monomorium chinense* workers and α-neocallitropsene (b) from the literature book, showing the match of mass spectra.

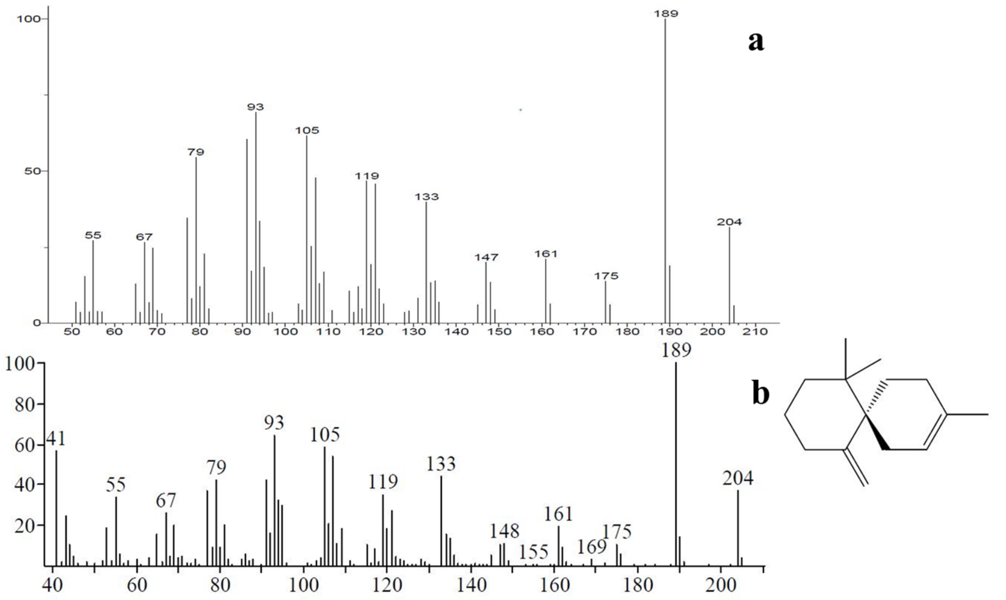

Figure S7 Mass spectra of peak 7 (a) from *Monomorium chinense* workers and β-chamigrene (b) from the literature ~~book~~, showing the match of mass spectra.

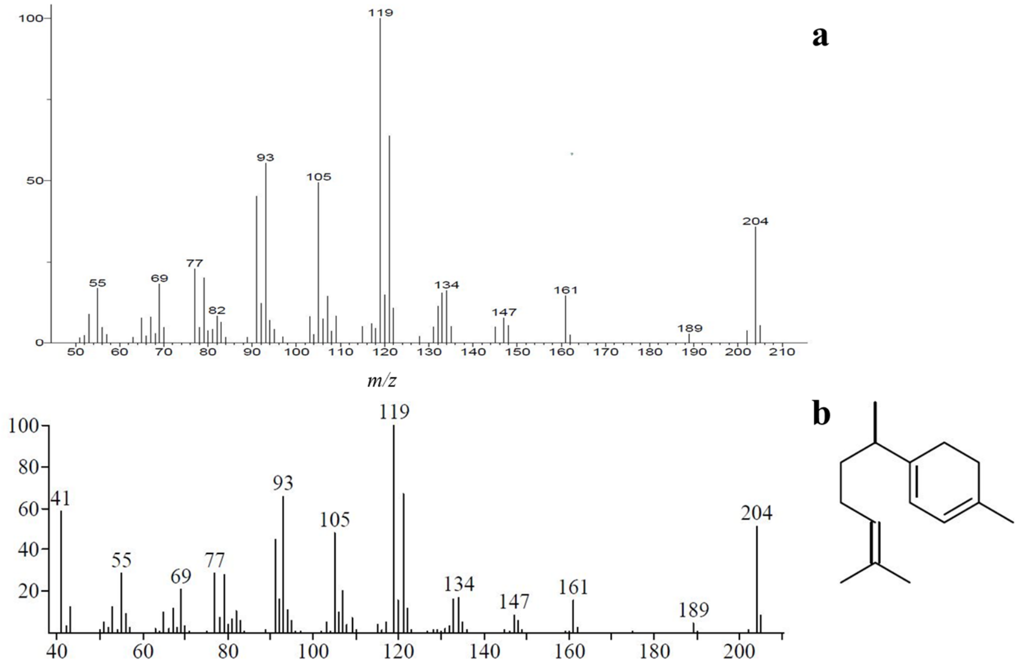

Figure S8 Mass spectra of peak 8 (a) from *Monomorium chinense* workers and γ-curcumene (b) from the literature ~~book~~, showing the match of mass spectra.

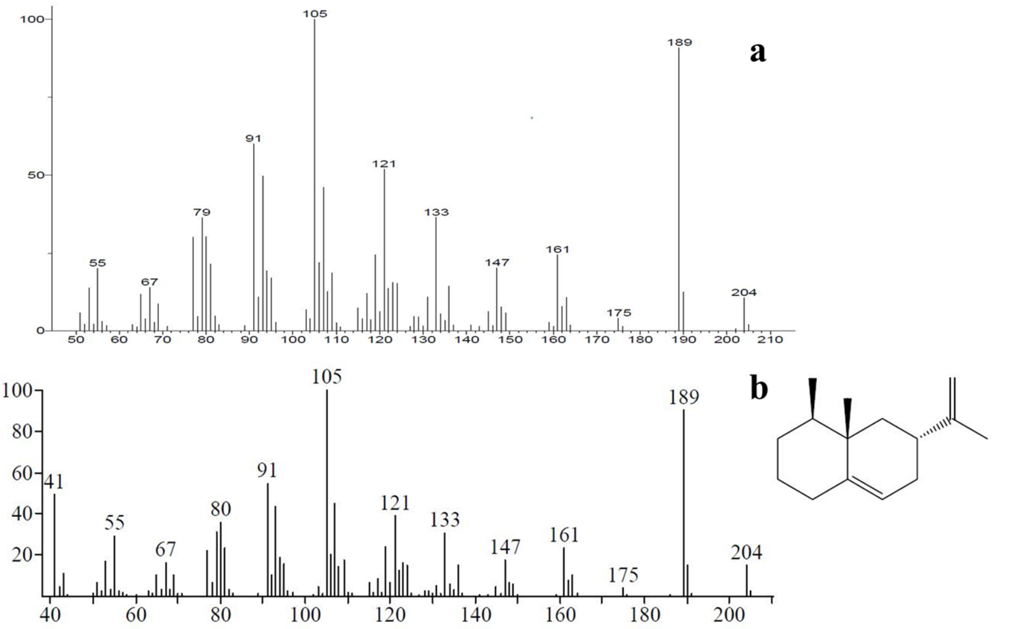

Figure S9 Mass spectra of peak 9 (~~a~~) from *Monomorium chinense* workers and aristolochene (~~b~~) from the literature ~~book~~, showing the match of mass spectra.

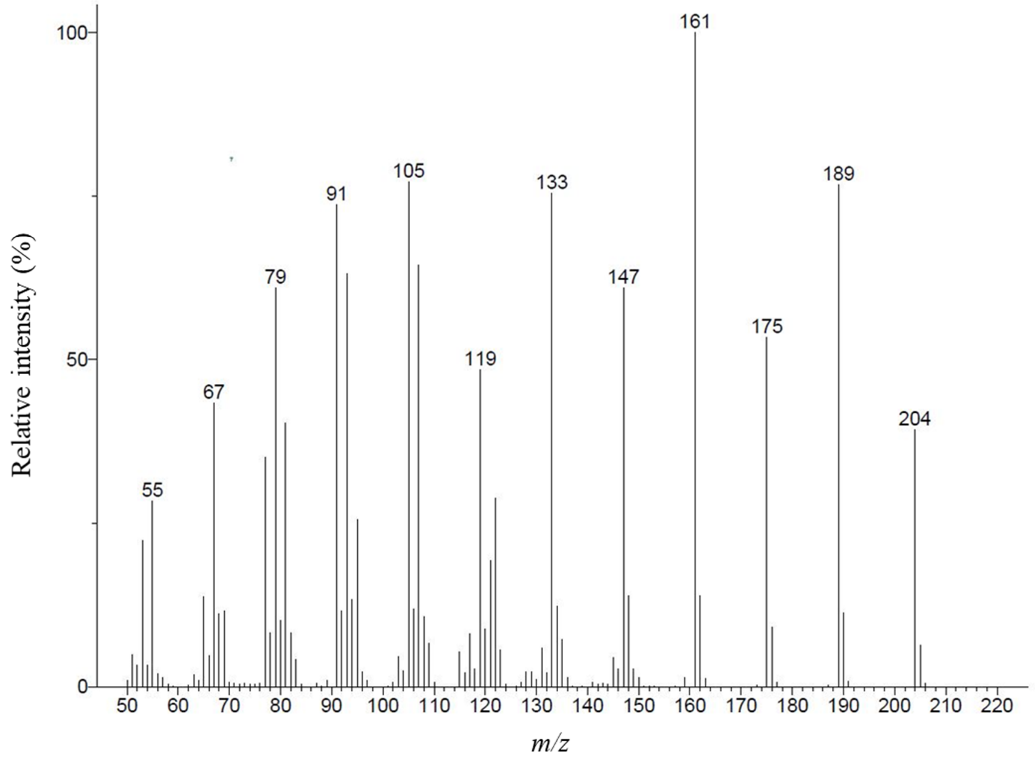

Figure S10 Mass spectrum of p10 from *Monomorium chinense* workers

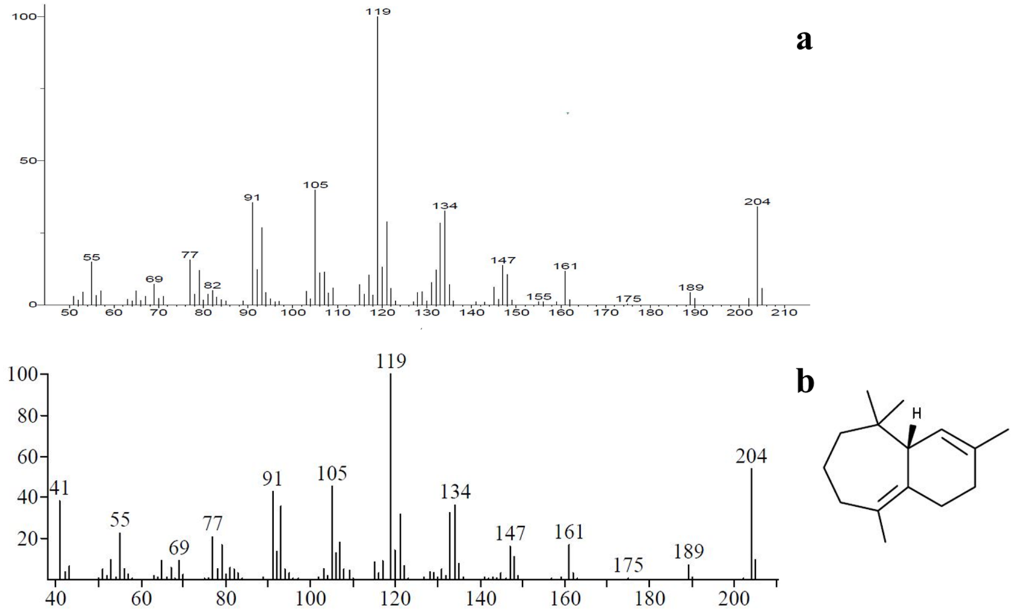

Figure S11 Mass spectra of peak 11 (a) from *Monomorium chinense* workers and β-himachalene (b) from the literature ~~book~~, showing the match of mass spectra.

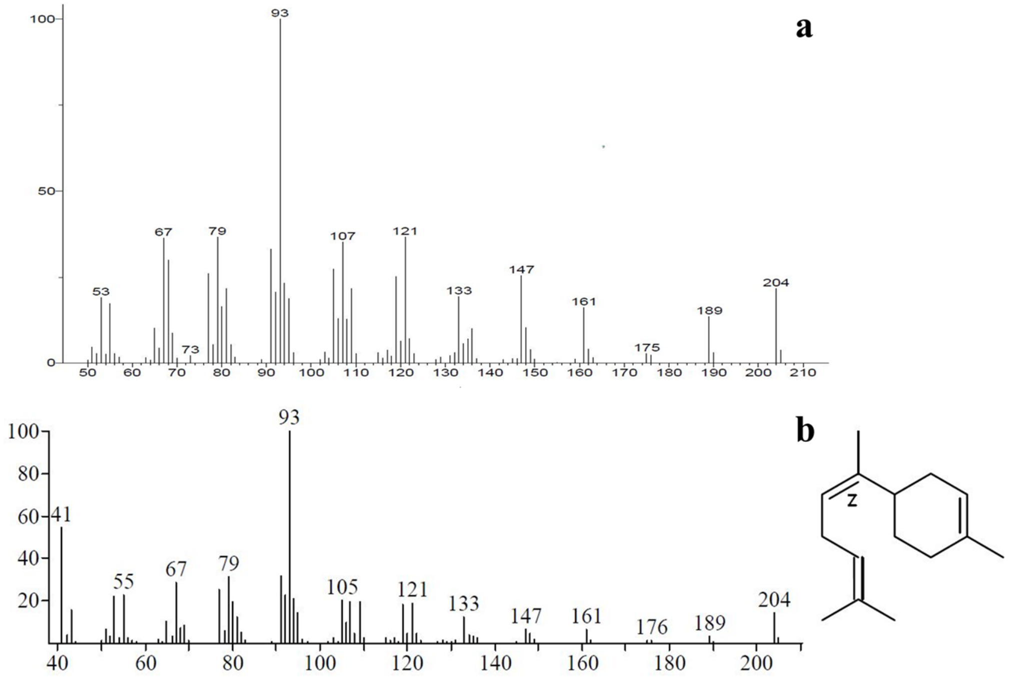

Figure S12 Mass spectra of peak 12 (a) from *Monomorium chinense* workers and (Z)-α-bisabolene (b) from the literature ~~book~~, showing the match of mass spectra.

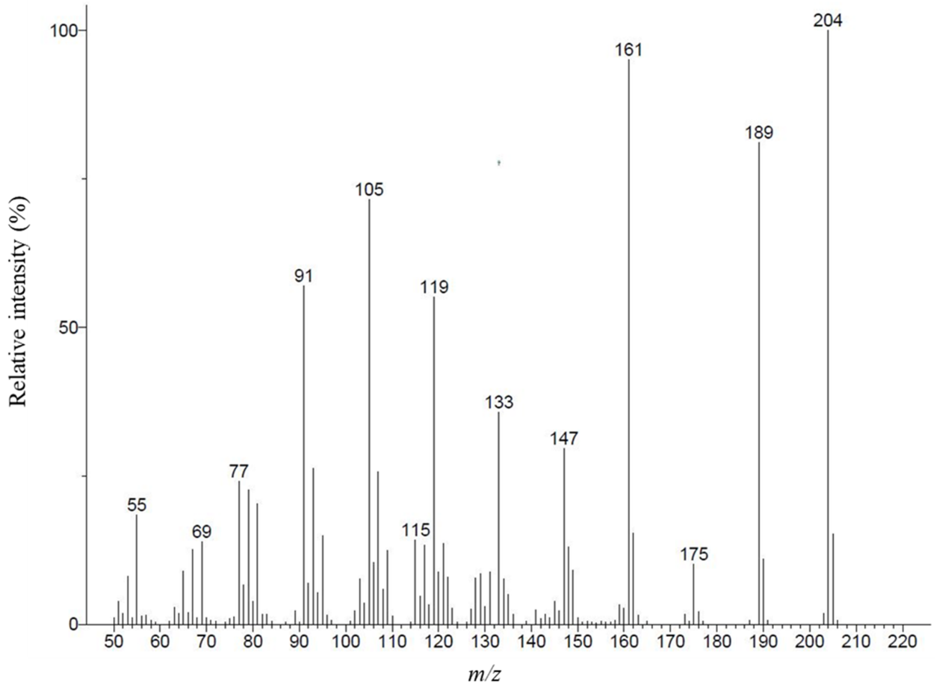

Figure S13 Mass spectrum of p13 from *Monomorium chinense* workers

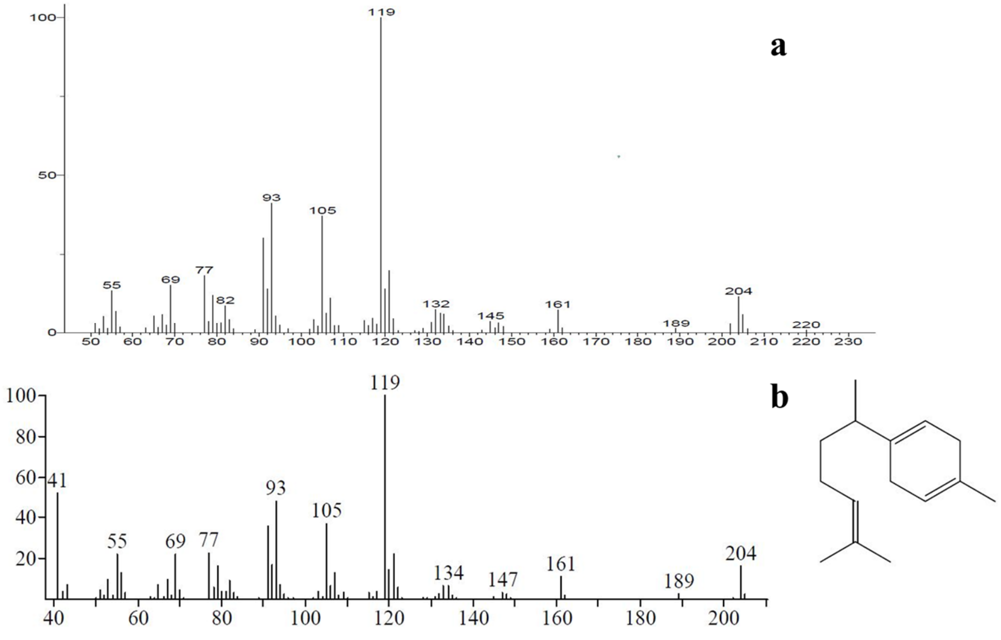

Figure S14 Mass spectra of peak 14 (a) from *Monomorium chinense* workers and β-curcumene (b) from the literature ~~book~~, showing the match of mass spectra.

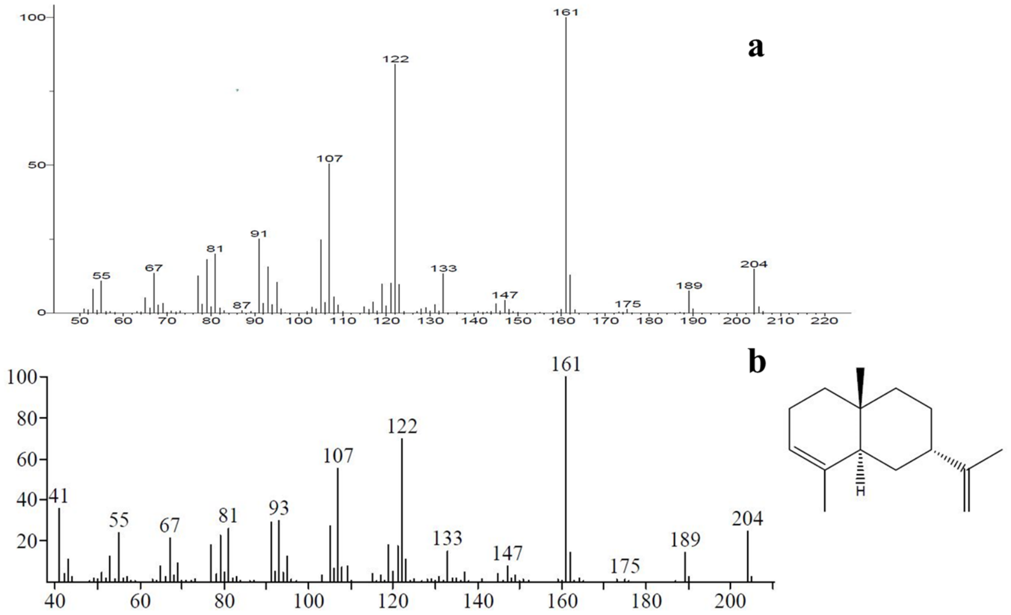

Figure S15 Mass spectra of peak 15 (a) from *Monomorium chinense* workers and 7-epi-α-selinene (b) from the literature ~~book~~, showing the match of mass spectra.

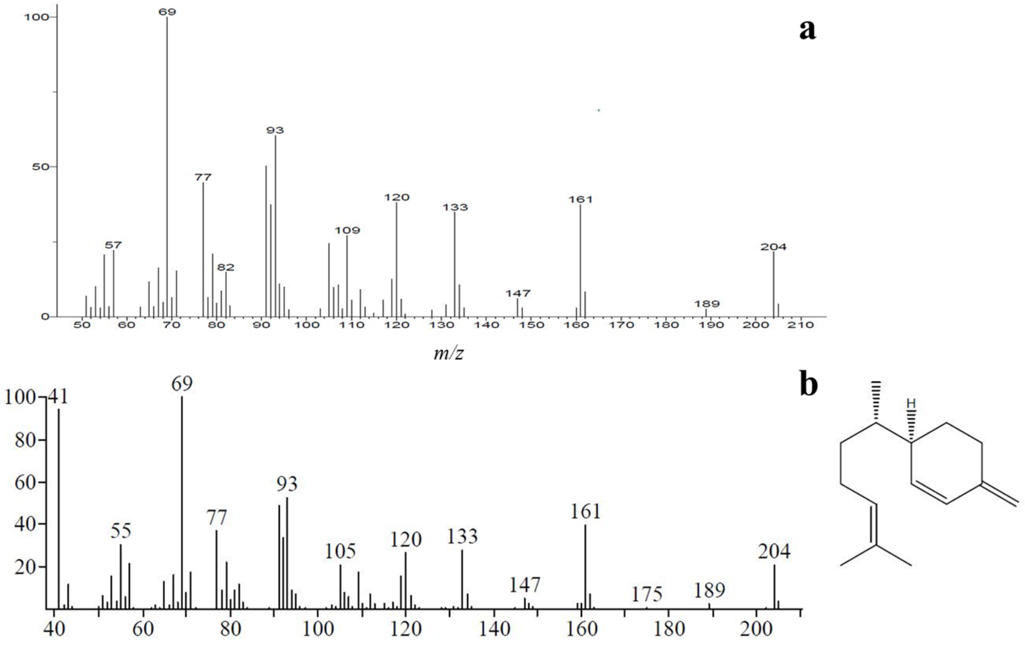

Figure S16 Mass spectra of peak 16 (a) from *Monomorium chinense* workers and β-sesquiphellandrene (b) from the literature ~~book~~, showing the match of mass spectra.

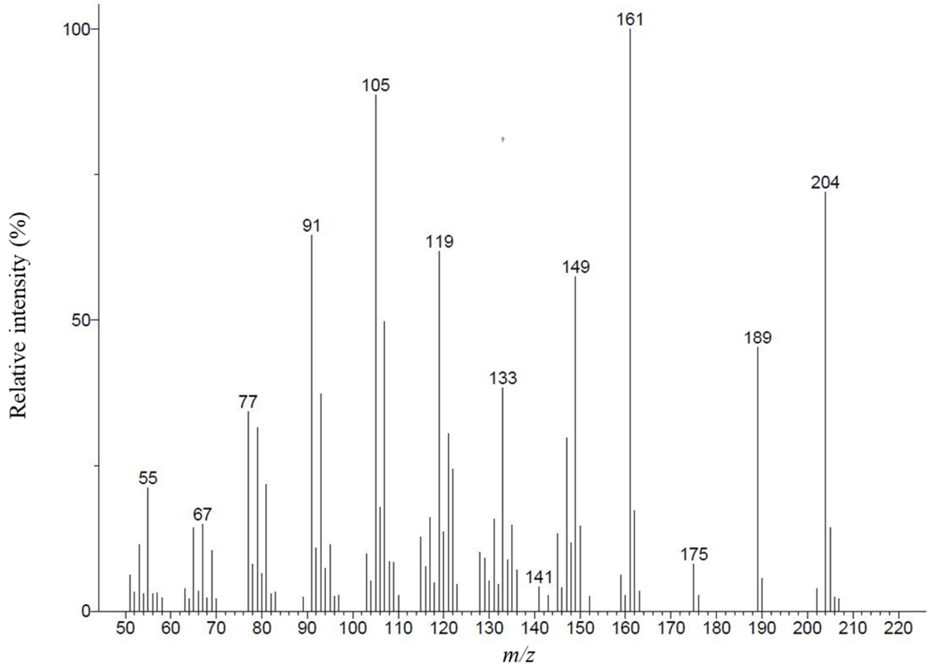

Figure S17 Mass spectrum of p17 from *Monomorium chinense* workers

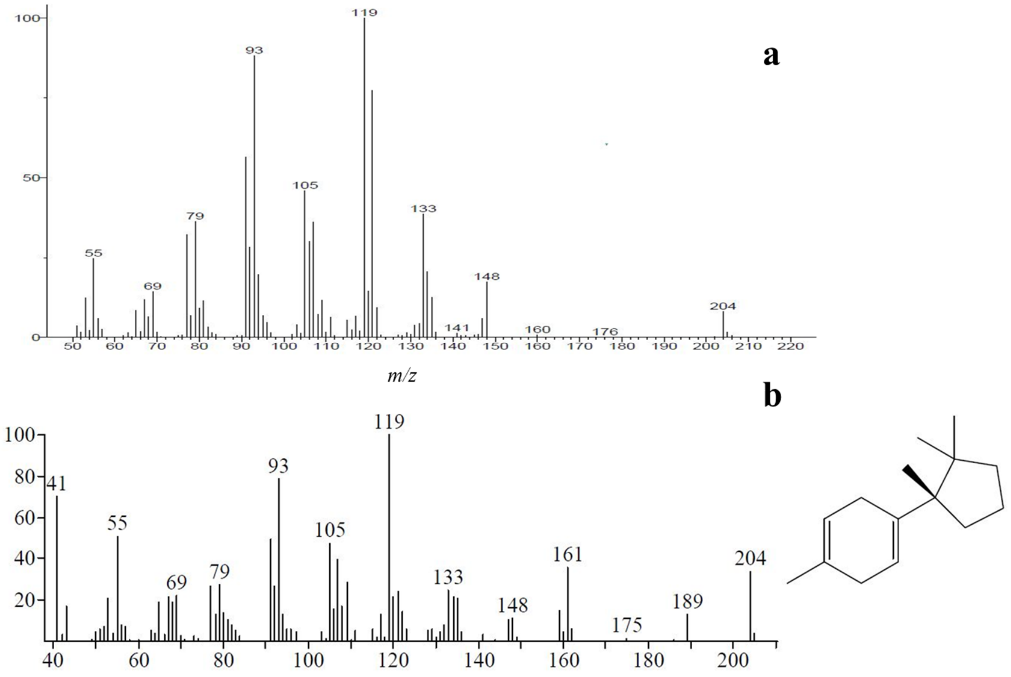

Figure S18 Mass spectra of peak 18 (a) from *Monomorium chinense* workers ~~whole body extraction~~ and γ-cuprenene (b) from the literature ~~book~~, showing the match of mass spectra.

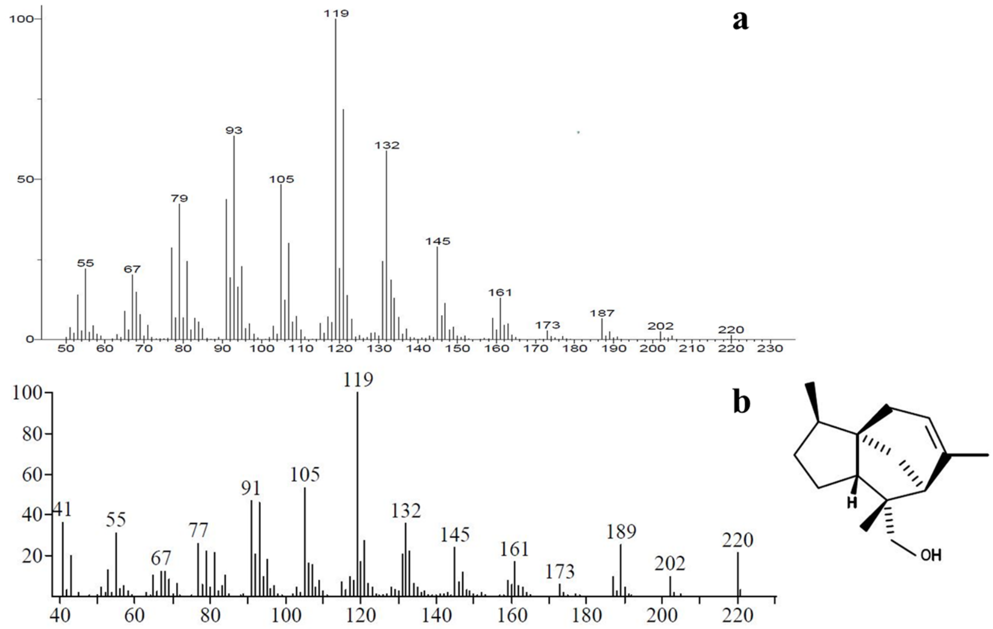

Figure S19 Mass spectra of peak 19 (a) from *Monomorium chinense* workers and 8-cedren-13-ol (b) from the literature book, showing the match of mass spectra.

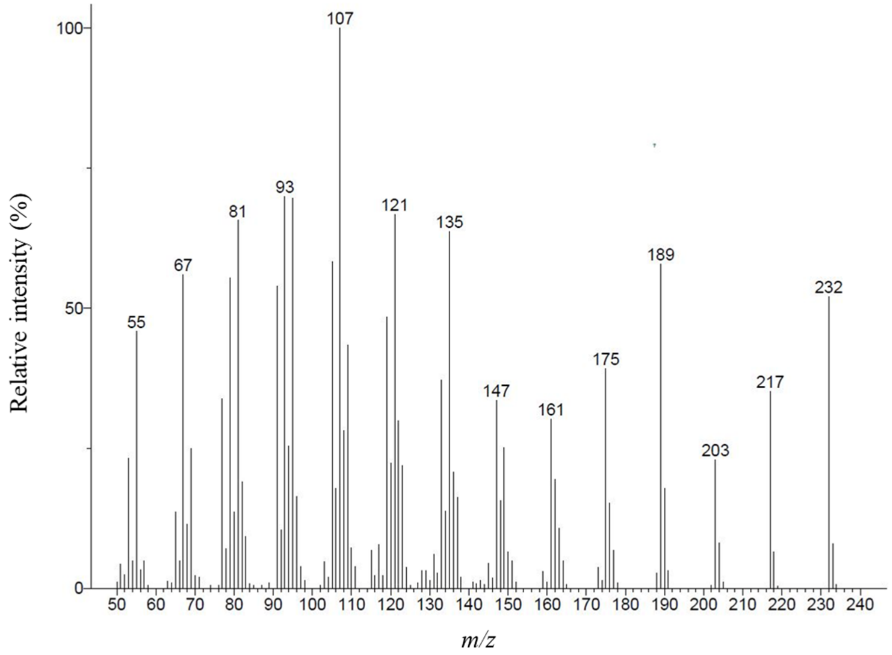

Figure S20 Mass spectrum of p20 from *Monomorium chinense* workers

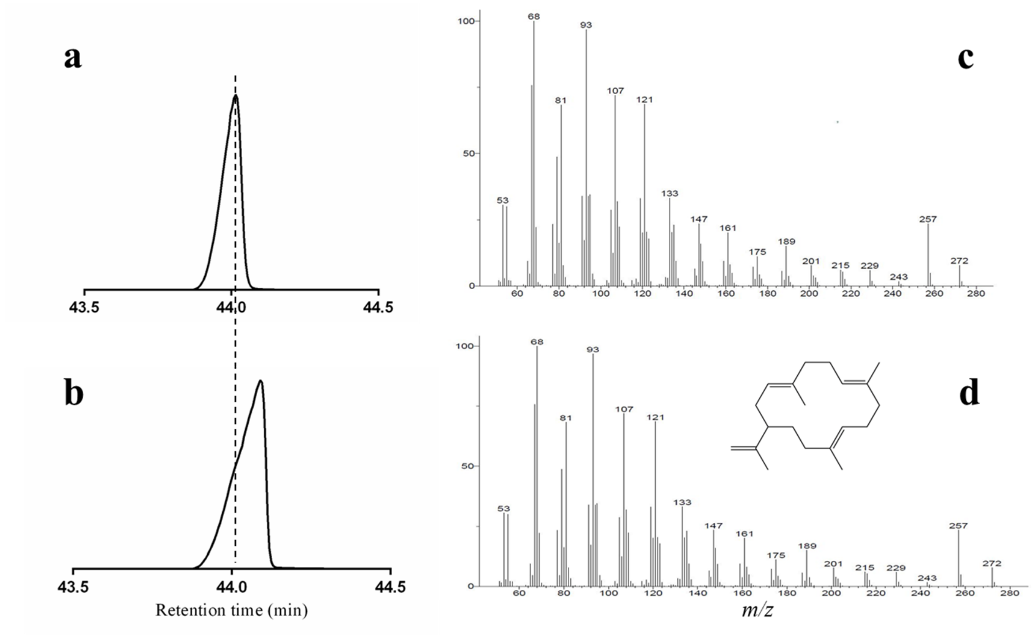

Figure S21 Total ion chromatograms of extract of *Monomorium chinense* workers (a) and *Monomorium pharaonis* workers (b), showing the match of retention times; Mass spectra of peak 2(c) and neocembrene (d), showing the match of mass spectra.

Table S1 Terpenes and terpenoids in insects

Order	Family	Common name	Number	Reference
Blattodea	Blattidae	Cockroach	2	[1,2]
Coleoptera	Carabidae	Ground beetle	15	[3]
	Chrysomelidae	Leaf beetle	10	[4-6]
	Scolytidae	Bark beetle	15	[7-12]
	Meloidae	Blister beetle	1	[13]
	Oedemeridae	False blister beetle	1	[13]
	Staphylinidae	Rove beetle	18	[14-17]
	Silphidae	Carrion beetle	4	[18,19]
	Tenebrionidae	Darkling beetle	4	[20]
Diptera	Psychodidae	Moth fly, Sand fly	3	[16,21]
	Tephritidae	Fruit fly	12	[22-24]
Heteroptera	Pentatomidae	Stink bug	5	[25,26]
	Pyrrhocoridae	Red bug	4	[27,28]
	Rhopalidae	Scentless plant bug	10	[29]
	Lygaeidae	Seed bug	3	[30]
Homoptera	Aphididae	Aphid	20	[31-33]
Hymenoptera	Tenthredinidae	Sawfly	4	[34]
	Formicidae	Ant	50	[35-53]
	Andrenidae	Mining bee	16	[54-56]
	Apidae	Honey bee	31	[57-63]
Isoptera	Rhinotermitidae	Termite	53	[53,64-70]
	Termitidae	Termite	30	[71-80]
	Nasutitermitidae	Termite	47	[81-92]
Lepidoptera	Papilionidae	Swallowtail butterfly	60	[93-100]
	Pieridae	Butterfly	11	[101-104]
	Pyralidae	Pyralid moth	2	[105]
	Nymphalidae	Brush-footed butterfly	3	[106,107]
Phasmatodea	Pseudophasmatidae	Stick insect	3	[108,109]
	Phasmatidae	Striped walkingstick	3	[110]
	Diapheromeridae	Stick insect	1	[111]
Total			220	

Table S2 Terpenes and terpenoids in ants and their glandular source

Category	Terpene	Subfamily	Gland	Reference
Monoterpene	Citral	Formicinae	Mandibular gland	[112]
	Citronellal	Formicinae	Rectum	[113]
	Citronellol	Formicinae, Myrmicinae	Rectum, Mandibular gland	[37,113]
	Geranial	Formicinae, Myrmicinae	Mandibular gland	[38,114]
	Geraniol	Myrmicinae	Mandibular gland	[38]
	4-Methylgeraniol	Ponerinae	Dufour's gland	[39]
	Bishomogeraniol	Ponerinae	Dufour's gland	[39]
	α-Pinene	Formicinae, Myrmicinae, Dolichoderinae	Rectum, Dufour's gland	[40,113]
	β-Pinene	Formicinae, Myrmicinae	Rectum, Dufour's gland	[40,113]
	Myrcene	Formicinae, Myrmicinae, Dolichoderinae	Rectum	[113]
	Cymene	Formicinae, Myrmicinae, Dolichoderinae	Rectum	[113]
	Limonene	Formicinae, Myrmicinae, Dorylinae, Dolichoderinae	Rectum, Mandibular gland, Poison gland	[41,113,115]
	Camphor	Formicinae	Rectum	[113]
	Camphene	Myrmicinae	Rectum,Dufour's gland	[40,113]
	β-Terpinene	Myrmicinae	Dufour's gland	[40]
	γ-Terpinene	Myrmicinae	Dufour's gland	[40]
	α-Terpinolene	Myrmicinae	Dufour's gland	[40]
	Neral	Myrmicinae	Mandibular gland	[42]

	Lasiol,	Formicinae	Mandibular gland	[44]
	β-Ocimene	Ecitoninae	Dufour's gland	[116]
	Iridodial	Dolichoderinae	Pygidial gland	[43]
	α-Farnesene	Formicinae, Myrmicinae, Nothomyrmeciinae	Mandibular gland, Dufour's gland	[35,45-48,114]
	β-Farnesene	Myrmicinae	Dufour's gland	[35,47]
	α-Homofarnesene	Myrmicinae	Dufour's gland	[47,49]
Sesquiterpene	Bishomofarnesene	Myrmicinae	Dufour's gland	[47,49]
	Homofarnesene	Myrmicinae, Formicinae	Dufour's gland	[45,50]
	Trishomofarnesene	Myrmicinae	Dufour's gland	[35]
	β-Elemene	Myrmicinae	Venom gland	[36]
	β-Springene	Myrmicinae	Dufour's gland	[51]
	Geranyl geranial	Formicinae, Dorylinae	Mandibular gland, Dufour's gland	[41,117]
	Geranylgeraniol	Formicinae	Dufour's gland	[118]
Diterpene	Geranyl citronellal	Formicinae	Dufour's gland	[117]
	Geranyl linalool	Formicinae	Dufour's gland	[52]
	Geranylfarnesol	Formicinae	Dufour's gland	[52]
	Neocembrene	Myrmicinae	Dufour's gland	[51,119]

References:

1. Kuwahara, S.; Mori, K. Synthesis of both the enantiomers of Hauptmann's periplanone-A and clarification of the structure of Persoons's periplanone-A. *Tetrahedron* **1990**, 46(24), 8083-8092; DOI: 10.1002/chin.199111335.
2. Kuwahara, S.; Mori, K. Synthesis of (-)-periplanone-B a sex pheromone component of the American cockroach (*Periplaneta Americana*). *Tetrahedron* **1990**, 46(24), 8075-8082; DOI: 10.1016/S0040-4020(01)81464-2.
3. Giglio, A.; Brandmayr, P.; Dalpozzo, R.; Sindona, G.; Tagarelli, A.; Talarico, F.; Brandmayr, T.Z.; Ferrero, E.A. The defensive secretion of *Carabus lefebvrei* Dejean 1826 pupa (Coleoptera, Carabidae): gland ultrastructure and chemical identification. *Microsc. Res. Tech*. **2009**, 72(5), 351-361; DOI: 10.1002/jemt.20660.
4. Bartelt, R.J.; Cossé, A.A.; Zilkowski, B.W.; Weisleder, D.; Momany, F.A. Male-specific sesquiterpenes from *Phyllotreta* and *Aphthona* flea beetles. *J. Chem. Ecol.* **2001**, 27(12), 2397-2423; DOI: 10.1023/A:1013667229345.
5. Evans, P.H.; Becerra, J.X.; Venable, D.L.; Bowers, W.S. Chemical analysis of squirt-gun defense in *Bursera* and counterdefense by Chrysomelid beetles. *J. Chem. Ecol.* **2000**, 26(3), 745-754; DOI: 10.1023/A:1005436523770.
6. Beran, F.; Jiménez-Alemán, G.H.; Lin, M.Y.; Hsu, Y.C.; Mewis, I.; Srinivasan, R.; Ulrichs, C.; Boland, W.; Hansson, B.S.; Reinecke, A. The aggregation pheromone of *Phyllotreta striolata* (Coleoptera: Chrysomelidae) revisited. *J. Chem. Ecol.* **2016**, 42(8), 748-755; DOI: 10.1007/s10886-016-0743-6.
7. Kohnle, U.; Schmutzenhofer, H.; Bartels, J.; Francke, W. Oxygenated terpenes in the chemical communication system of the bark beetle, *Ips schmutzenhoferi* (Col., Scolytidae), a species recently described for the Southeastern Himalaya. *J. Appl. Entomol.* **1988**, 106(1-5), 46-51; DOI: 10.1111/j.1439-0418. 1988.tb00562.x.
8. Zuber, M.; Meyer, H.; Kohnle, U.; Francke, W. Odour production and pheromone response

in the European engraver bark beetles, *Ips amitinus* (Eichh.) and *Ips amitinus* var. *montana* Fuchs (Col., Scolytidae). *J. Appl. Entomol.* **1993**, ~~115(1-5)~~, 462-465~~;~~ DOI: 10.1111/j.1439-0418.1993.tb00415.x.

9. Seybold, S.J.; Ohtsuka, T.; Wood, D.L.; Kubo, I. Enantiomeric composition of ipsdienol: a chemotaxonomic character for North American populations of *Ips spp.* in the pini subgeneric group (Coleoptera: Scolytidae). *J. Chem. Ecol.* **1995**, ~~21(7)~~, 995-1016~~;~~ DOI: 10.1007/BF02033804.
10. Kohnle, U.; Pajares, J.A.; Bartels, J.; Meyer, H.; Francke, W. Chemical communication in the European pine engraver, *Ips mannsfeldi* (Wachtl) (Col., Scolytidae). *J. Appl. Entomol.* **1993**, ~~115(1-5)~~, 1-7~~;~~ DOI: 10.1111/j.1439-0418. 1993.tb00357.x.
11. Birgersson, G.; Debarr, G.L.; Groot, P.D.; Dalusky, M.J.; Jr, H.D.P.; Borden, J.H.; Meyer, H.; Francke, W.; Espelie, K.E.; Berisford, C.W. Pheromones in white pine cone beetle, *Conophthorus coniperda* (schwarz) (Coleoptera: Scolytidae). *J. Chem. Ecol.* **1995**, ~~21(2)~~, 143-167~~;~~ DOI: 10.1007/BF02036648.
12. Francisco A. Marques; Scheila R. M. Zaleski; Sonia M. N. Lazzari; Gustavo Frensch; Grece A. Senhorini; Beatriz H. L. N. S. Maia;Armin Tröger; Wittko Francke; Edson T. Ieded ; Kenji Morie. Identification of (1~~R~~, 2~~S~~)-grandisal and (1~~R~~, 2~~S~~)-grandisol in *Pissodes castaneus* male-produced volatiles: evidence of a sex pheromone. *J. Braz. Chem. Soc.* **2011**, ~~22(6)~~, 1050-1055~~;~~ DOI: 10.1590/S0103-50532011000600007.
13. Hashimoto, K.; Hayashi, F. Cantharidin world in nature: a concealed arthropod assemblage with interactions via the terpenoid cantharidin. *Entomol. Sci.* **2015**, ~~17(4)~~, 388-395~~;~~ DOI: 10.1111/ens.12074.
14. Weibel, D.B.; Oldham, N.J.; Feld, B.; Glombitza, G.; Dettner, K.; Boland, W. Iridoid biosynthesis in staphylinid rove beetles (Coleoptera: Staphylinidae, Philonthinae). *Insect Biochem. Mol. Biol.* **2001**, ~~31(6)~~, 583-591~~;~~ DOI: 10.1016/S0965-1748(00)00163-6.
15. Huth, A.; Dettner, K. Defense chemicals from abdominal glands of 13 rove beetle species

of subtribe Staphylinina (Coleoptera: Staphylinidae, Staphylininae). *J. Chem. Ecol.* **1990**, ~~16(9)~~, 2691-2711~~;~~ DOI: 10.1007/BF00988079.

16. Müller, M.; Buchbauer, G. Essential oil components as pheromones. A review. *Flavour Fragr. J.* **2011**, ~~26(6)~~, 357-377; DOI: 10.1002/ffj.2055.

17. Bellas, T.E.; Brown, W.V.; Moore, B.P. The alkaloid actinidine and plausible precursors in defensive secretions of rove beetles. *J. Insect Physiol.* **1974**, ~~20(2)~~, 277-280~~;~~ DOI: 10.1016/0022-1910(74)90059-6.

18. Eisner, T.; Deyrup, M.; Jacobs, R.; Meinwald, J. Necrodols: anti-insectan terpenes from defensive secretion of carrion beetle (*Necrodes surinamensis*). *J. Chem. Ecol.* **1986**, ~~12(6)~~, 1407-15~~;~~ DOI: 10.1007/BF01012360.

19. Haberer, W.; Schmitt, T.; Schreier, P.; Eggert, A.K.; Müller, J.K. Volatiles emitted by calling males of burying beetles and *Ptomascopus morio* (Coleoptera: Silphidae: Nicrophorinae) are biogenetically related. *J. Chem. Ecol.* **2017**, ~~43(11-12)~~, 1126-1127~~;~~ DOI: 10.1007/s10886-017-0892-2.

20. Zilkowski, B.W.; Bartelt, R.J.; Cossé, A.A.; Petroski, R.J. Male-produced aggregation pheromone compounds from the eggplant flea beetle (*Epitrix fuscula*): identification, synthesis, and field biossays. *J. Chem. Ecol.* **2006**, ~~32(11)~~, 2543-2558~~;~~ DOI: 10.1007/s10886-006-9163-3.

21. Hamilton, J.G.C.; Dawson, G.W.; Pickett, J.A. 9-Methylgermacrene-B; proposed structure for novel homosesquiterpene from the sex pheromone glands of *Lutzomyia longipalpis* (Diptera: Psychodidae) from Lapinha, Brazil. *J. Chem. Ecol.* **1996**, ~~22(8)~~, 1477-1491~~;~~ DOI: 10.1007/BF02027726.

22. Rocca, J.R.; Nation, J.L.; Strekowski, L.; Battiste, M.A. Comparison of volatiles emitted by male caribbean and mexican fruit flies. *J. Chem. Ecol.* **1992**, ~~18(2)~~, 223-44~~;~~ DOI: 10.1007/BF00993755.

23. Baker, R.; Herbert, R.H.; Grant, G.G. Isolation and identification of the sex pheromone of the mediterranean fruit fly, *Ceratitis capitate* (Wied). *Chem. Comm.* **1985**, ~~12(12)~~, 824-

825; DOI: 10.1039/C39850000824.

24. Raptopoulos, D.; Haniotakis, G.; Koutsaftikis, A.; Kelly, D.; Mavraganis, V. Biological activity of chemicals identified from extracts and volatiles of male *Rhagoletis cerasi*. *J. Chem. Ecol.* **1995**, ~~21(9)~~, 1287-97; DOI: 10.1007/BF02027562.

25. Brézot, P.; Malosse, C.; Mori, K.; Renou, M. Bisabolene epoxides in sex pheromone in *Nezara viridula* (L.) (Heteroptera: Pentatomidae): role of *cis* isomer and relation to specificity of pheromone. *J. Chem. Ecol.* **1994**, ~~20(12)~~, 3133-3147; DOI: 10.1007/BF02033716.

26. Mcbrien, H.L.; Millar, J.G.; Rice, R.E.; Mcelfresh, J.S.; Cullen, E.; Zalom, F.G. Sex attractant pheromone of the red-shouldered stink bug *Thyanta pallidovirens*: a pheromone blend with multiple redundant components. *J. Chem. Ecol.* **2002**, ~~28(9)~~, 1797-1818; DOI: 10.1023/A:102051321.

27. Farine, J.P.; Bonnard, O.; Brossut, R.; Quere, J.L.L. Chemistry of pheromonal and defensive secretions in the nymphs and the adults of *Dysdercus cingulatus* Fabr. (Heteroptera, Pyrrhocoridae). *J. Chem. Ecol.* **1992**, ~~18(1)~~, 65; DOI: 10.1007/BF00997165.

28. Rudmann, A.A.; Aldrich, J.R. Chirality determinations for a tertiary alcohol: ratios of linalool enantiomers in insects and plants. *J. Chromatogr. A*. **1987**, ~~407(01)~~, 324-329; DOI: 10.1016/S0021-9673(01)92632-9.

29. Ho, H.Y.; Hsu, Y.C.; Ho, J.Z.; Lo, L.W.; Chuang, Y.C. Volatiles in the dorsal abdominal glands and exuviae of *Leptocoris abdominalis* and *Leptocoris augur* (Heteroptera: Rhopalidae). *Ann. Entomol. Soc. Am.* **2006**, ~~99(5)~~, 859-864; DOI: 10.1603/0013-8746(2006)99[859: VITDAG]2.0.CO;2.

30. Olagbemiro, T.O.; Staddon, B.W. Isoprenoids from the metathoracic scent gland of cotton seed bug, *Oxycarenus hyalinipennis* (Costa) (Heteroptera: Lygaeidae). *J. Chem. Ecol.* **1983**, ~~9(10)~~, 1397-1412; DOI: 10.1007/BF00990746.

31. Francis, F.; Vandermoten, S.; Verheggen, F.; Lognay, G.; Haubruge, E. Is the (E)-β-farnesene only volatile terpenoid in aphids? *J. Appl. Entomol.* **2010**, ~~129(1)~~, 6-11; DOI:

10.1111/j.1439-0418.2005. 00925.x.

32. Dawson, G.W.; Griffiths, D.C.; Merritt, L.A.; Mudd, A.; Pickett, J.A.; Wadhams, L.J.; Woodcock, C.M. Aphid semiochemicals - a review, and recent advances on the sex pheromone. *J. Chem. Ecol.* **1990**, ~~16(11)~~, 3019-3030~~;~~ DOI: 10.1007/BF00979609.

33. Goldansaz, S. H.; Dewhirst, S.; Birkett, M. A.; Hooper, A. M.; Smiley, D. W.; Pickett, J. A.; Wadhams, L.; McNeil, J. N. Identification of two sex pheromone components of the potato aphid, *Macrosiphum euphorbiae* (Thomas). *J. Chem. Ecol.* **2004**, ~~30(4)~~, 819-834~~;~~ DOI: 10.1023/B:JOEC.0000028434.19319.b4.

34. Boeve, J.L.; Dettner, K.; Francke, W.; Meyer, H.; Pasteels, J.M. The secretion of the ventral glands in *Hoplocamp* sawfly larvae. *Biochem. Syst. Ecol.* **1992**, ~~25(20)~~, 107-111~~;~~ DOI: 10.1016/0305-1978(92)90097-W.

35. Ali, M.F.; Jackson, B.D.; Morgan, E.D. Contents of the poison apparatus of some species of *Pheidole* ants. *Biochem. Syst. Ecol.* **2007**, ~~35(10)~~, 641-651~~;~~ DOI: 10.1016/j.bse.2007.03.025.

36. Cruz-López, L.; Rojas, J.C.; De, L.C.R.; Morgan, E.D. Behavioral and chemical analysis of venom gland secretion of queens of the ant *Solenopsis geminata*. *J. Chem. Ecol.* **2001**, ~~27(12)~~, 2437~~-45;~~ DOI: 10.1023/A:1013671330253.

37. Keegans, S.J.; Morgan, E.D.; Agosti, D.; Wehner, R. What do glands tell us about species? A chemical case study of *Cataglyphis* ants. *Biochem. Syst. Ecol.* **1992**, ~~20(6)~~, 559-572~~;~~ DOI: 10.1016/0305-1978(92)90010-B.

38. Blum, M.S.; Padovani, F.; Amante, E. Alkanones and terpenes in the mandibular glands of *Atta* species (Hymenoptera: Formicidae). *Comp. Biochem. Physiol. B.* **1968**, ~~26(1)~~, 291-299~~;~~ DOI: 10.1016/0010-406X(68)90333-2.

39. Schulz, C.M.; Lehmann, L.; Blatrix, R.; Jaisson, P.; Hefetz, A.; Francke, W. Identification of new homoterpene esters from Dufour's gland of the ponerine ant *Gnamptogenys striatula*. *J. Chem. Ecol.* **2002**, ~~28(12)~~, 2541~~-55;~~ DOI: 10.1023/A:1021492204400.

40. Billen, J.; Ito, F.; Tsuji, K.; Schoeters, E.; Maile, R.; Morgan, E.D. Structure and chemistry

of the Dufour gland in *Pristomyrmex* ants (Hymenoptera, Formicidae). *Acta Zool.* **2000**, ~~81(2)~~, 159-166~~;~~ DOI: 10.1046/j.1463-6395.2000. 00046.x.

41. Oldham, N.J.; Morgan, E.D.; Gobin, B.; Schoeters, E.; Billen, J. Volatile secretions of old world army ant *Aenictus rotundatus* and chemotaxonomic implications of army ant Dufour gland chemistry. *J. Chem. Ecol.* **1994**, ~~20(12)~~, 3297-3305~~;~~ DOI: 10.1007/BF02033727.
42. Francelino, M.R.; Mendonça, A.L.; Do Nascimento, R.R.; Sant'Ana, A.E. The mandibular gland secretions of the leaf-cutting ants *Atta sexdens sexdens* and *Atta opaciceps* exhibit intercaste and intercolony variations. *J. Chem. Ecol.* **2006**, ~~32(3)~~, 643~~;~~ DOI: 10.1007/s10886-005-9020-9.
43. Shi, Q.; Lu, L.; Lei, Y.; He, Y.; Chen, J. Gland origin and electroantennogram activity of volatile compounds in ghost Ants, *Tapinoma melanocephalum* (Hymenoptera: Formicidae) and behavioral response to (Z)-9-nonadecene. *Environ. Entomol.* **2017**, ~~46(6);~~ DOI: 10.1093/ee/nvx164.
44. Lloyd, H.A.; Jones, T.H.; Hefetz, A.; Tengö, J. Lasiol, a new acyclic monoterpenol in the mandibular gland secretion of *lasius meridionalis*. *Tetrahedron Lett.* **1990**, ~~31(39)~~, 5559-5562~~;~~ DOI: 10.1016/S0040-4039(00)97896-1.
45. Ali, M.F.; Attygalle, A.B.; Morgan, E.D.; Billen, J.P.J. The Dufour gland substances of the workers of *Formica fusca* and *Formica lemani* (Hymenoptera: Formicidae). *Comp. Biochem. Physiol. B.* **1987**, ~~88(1)~~, 59-63~~;~~ DOI: 10.1016/0305-0491(87)90079-4.
46. Cavill, G.W.K.; Williams, P.J.; Whitfield, F.B. α-Farnesene, Dufour's gland secretion in the ant *Aphaenogaster longiceps*. *Tetrahedron Lett.* **1967**, ~~8(23)~~, 2201-2205~~;~~ DOI: 10.1016/S0040-4039(00)90797-4.
47. Jackson, B.D.; Cammaerts, M.C.; Morgan, E.D.; Attygalle, A.B. Chemical and behavioral studies on Dufour gland contents of *Manica rubida* (Hymenoptera: Formicidae). *J. Chem. Ecol.* **1990**, ~~16(3)~~, 827~~;~~ DOI: 10.1007/BF01016493.
48. Cammaerts, M.C.; Evershed, R.P.; Morgan, E.D. Comparative study of the Dufour gland secretions of workers of four species of *Myrmica* ants. *J. Insect Physiol.* **1981**, ~~27(1)~~, 59-

65~~;~~ DOI: 10.1016/0022-1910(81)90033-0.

49. Attygalle, A.B.; Cammaerts, M.C.; Morgan, E.D. Dufour gland secretions of *Myrmica rugulosa* and *Myrmica schencki* workers. *J. Insect Physiol.* **1983**, ~~29(1)~~, 27-32~~;~~ DOI: 10.1016/0022-1910(83)90102-6.

50. Adams, R.M.M.; Jones, T.H.; Jeter, A.W.; De Fine Licht, H.H.; Schultz, T.R.; Nash, D.R. A comparative study of exocrine gland chemistry in *Trachymyrmex* and *Sericomyrmex* fungus-growing ants. *Biochem. Syst. Ecol.* **2012**, ~~40~~, 91-97~~;~~ DOI: 10.1016/j.bse.2011.10.011.

51. Chen, J.; Cantrell, C.L.; Oi, D.; Grodowitz, M.J. Update on the defensive chemicals of the little black ant, *Monomorium minimum* (Hymenoptera: Formicidae). *Toxicon* 2016, ~~122~~, 127-132~~;~~ DOI: 10.1016/j.toxicon.2016.09.009.

52. Brand, J.M.; Mabinya, L.V.; Morgan, E.D. Volatile chemicals in glands of the carpenter ant, *Camponotus arminius*. *Afr. Zool.* **1999**, ~~34(3)~~, 140-142~~;~~ DOI: 10.1080/02541858.1999.11448500.

53. Chen, J. Freeze-thaw sample preparation method improves detection of volatile compounds in insects using headspace solid-phase microextraction. *Anal. Chem.* **2017**, ~~89(16)~~, 8366~~;~~ DOI: 10.1021/acs.analchem.7b01622.

54. Cane, J.H. Preliminary chemosystematics of the Andrenidae and exocrine lipid evolution of the short-tongued bees (Hymenoptera: Apoidea). *Syst. Biol.* **1983**, ~~32(4)~~, 417-430~~;~~ DOI: 10.2307/2413168.

55. Duffield, R.M.; Harrison, S.E.; Maglott, D.; Ayorinde, F.O.;Wheeler, J.W. Exocrine secretions of bees V. Terpenoid esters in the Dufour's secretions of *Panurginus* bees (Hymenoptera: Andrenidae). *J. Chem. Ecol.* **1983**, ~~9(2)~~, 277-283~~;~~ DOI: 10.1007/BF00988045.

56. Hefetz, A.; Eickwort, G.C.; Blum, M.S.; Cane, J.; Bohart, G.E. A comparative study of the exocrine products of cleptoparasitic bees (*Holcopasites*) and their hosts (*Calliopsis*) (Hymenoptera: Anthophoridae, Andrenidae). *J. Chem. Ecol.* **1982**, ~~8(11)~~, 1389~~-97;~~ DOI:

10.1007/BF01403102.

57. Pianaro, A.; Menezes, C.; Kerr, W.E.; Singer, R.B.; Patricio, E.F.; Marsaioli, A.J. Stingless bees: chemical differences and potential functions in *Nannotrigona testaceicornis* and *Plebeia droryana* males and workers. *J. Chem. Ecol.* **2009**, ~~35(9)~~, 1117-1128~~;~~ DOI: 10.1007/s10886-009-9679-4.
58. Cruz-López, L.; Rojas, J.C.; De, L.C.R.; Morgan, E.D. Behavioral and chemical analysis of venom gland secretion of queens of the ant *Solenopsis geminata*. *J. Chem. Ecol.* **2001**, ~~27(12)~~, 2437~~-45;~~ DOI: 10.1023/A:1013671330253.
59. Cassier, P.; Lensky, Y. The Nassanov gland of the workers of the honeybee (*Apis mellifera* L.): ultrastructure and behavioural function of the terpenoid and protein components. *J. Insect Physiol.* **1994**, ~~40(7)~~, 577-584~~;~~ DOI: 10.1016/0022-1910(94)90144-9.
60. Bergman, P.; Bergström, G. Scent marking, scent origin, and species specificity in male premating behavior of two Scandinavian bumblebees. *J. Chem. Ecol.* **1997**, ~~23(5)~~, 1235-1251~~;~~ DOI: 10.1023/B: JOEC.0000006461.69512.33.
61. Francke, W.; Krohn, S.; Tengö, J. Identification of new sesquiterpenoids in cephalic secretion of cuckoo bee, *Nomada lathburiana* (Apoidea, Anthophoridae). *J. Chem. Ecol.* **1991**, ~~17(3)~~, 557~~-66;~~ DOI: 10.1007/BF00982126.
62. Dewhirst, S.Y.; Birkett, M.A.; Fitzgerald, J.D.; Stewartjones, A.; Wadhams, L.J.; Woodcock, C.M.; Hardie, J.; Pickett, J.A. Dolichodial: a new aphid sex pheromone component? *J. Chem. Ecol.* **2008**, ~~34(12)~~, 1575-1583~~;~~ DOI: 10.1007/s10886-008-9561-9.
63. Blum, M.S.; Fales, H.M.; Morse, R.A.; Underwood, B.A. Chemical characters of two related species of giant honeybees (*Apis dorsata* and *A. laboriosa*): possible ecological significance. *J. Chem. Ecol.* **2000**, ~~26(4)~~, 801-807~~;~~ DOI: 10.1023/A:1005476405192.
64. Everaerts, C.; Roisin, Y.; Bonnard, O.; Pasteels, J.M. Sesquiterpenes in the frontal gland secretions of nasute soldier termites from New Guinea. *J. Chem. Ecol.* **1993**, ~~19(12)~~, 2865-~~79;~~ DOI: 10.1007/BF00980588.
65. Krasulová, J.; Hanus, R.; Kutalová, K., J, Š.; Sillam-Dussès, D.; Tichý, M.; Valterová, I.

Chemistry and anatomy of the frontal gland in soldiers of the sand termite *Psammotermes hybostoma*. *J. Chem. Ecol.* **2012**, ~~38(5)~~, 557-565~~;~~ DOI: 10.1007/s10886-012-0123-9.

66. Sobotnã K, J.; Hanus, R.; Kalinovã, B.; Piskorski, R.; Cvacka, J.; Bourguignon, T.; Roisin, Y. (E, E)-Alpha-farnesene, an alarm pheromone of the termite *Prorhinotermes canalifrons*. *J. Chem. Ecol.* **2008**, ~~34(4)~~, 478-486~~;~~ DOI: 10.1007/s10886-008-9450-2.

67. Quintana, A.; Reinhard, J.; Faure, R.; Uva, P.; Bagnères, A.G.; Massiot, G.; Clément, J.L. Interspecific variation in terpenoid composition of defensive secretions of European Reticulitermes termites. *J. Chem. Ecol.* **2003**, ~~29(3)~~, 639-652~~;~~ DOI: 10.1023/A:1022868603108.

68. Tarver, M.R.; Schmelz, E.A.; Rocca, J.R.; Scharf, M.E. Effects of soldier-derived terpenes on soldier caste differentiation in the termite *Reticulitermes flavipes*. *J. Chem. Ecol.* **2009**, ~~35(2)~~, 256-264~~;~~ DOI: 10.1007/s10886-009-9594-8.

69. Nguyen, T.T.; Kanaori, K.; Takematsu, Y.; Akino, T. Soldier-specific terpenoid compounds of the Japanese Reticulitermes (Isoptera, Rhinotermitidae). *Canadian Chemical Transactions* **2014**, ~~2(3)~~, 266-273~~;~~ DOI: :10.13179/canchemtrans.2014.02.03.0101.

70. Klochkov, S.G.; Kozlovskii, V.I.; Pushin, A.N. Isolation and identification of a trail attractant for the termite *Reticulitermes lucifugus* from the plant *Zizyphus jujuba*. *Chem. Nat. Compd.* **1989**, ~~25(3)~~, 361-363~~;~~ DOI: 10.1007/BF00597721.

71. Braekman, J.C.; Remacle, A.; Roisin, Y. Soldier defensive secretion of three Amitermes species. *Biochem. Syst. Ecol.* **1993**, ~~21(6-7)~~, 661-666~~;~~ DOI: 10.1016/0305-1978(93)90070-8.

72. Baker, R.; Evans, D.A.; Mcdowell, P.G. Mono- and sesquiterpenoid constituents of the defence secretion of the termite *Amitermes evuncifer*. *Tetrahedron Lett.* **1978**, ~~19(42)~~, 4073-4076~~;~~ DOI: 10.1016/S0040-4039(01)95143-3.

73. Moore, B.P. Volatile terpenes from Nasutitermes soldiers (Isoptera, Termitidae). *J. Insect Physiol.* **1964**, ~~10(2)~~, 371-375~~;~~ DOI: 10.1016/0022-1910(64)90020-4.

74. Bordereau, C.; Cancello, E.M.; Sillam-Dussès, D.; Sémon, E. Sex-pairing pheromones and

reproductive isolation in three sympatric *Cornitermes* species (Isoptera, Termitidae, Syntermitinae). *J. Insect Physiol.* **2011**, ~~57(4)~~, 469~~;~~ DOI: 10.1016/j.jinsphys.2011.01.010.

75. Prestwich, G. D. Interspecific variation of diterpene composition of *Cubitermes* soldier defense secretions. *J. Chem. Ecol.* **1984**, ~~10, (8)~~, 1219~~-31;~~ DOI: 10.1007/BF00988550.

76. Wiemer, D. F.; Meinwald, J.; Prestwich, G. D.; Solheim, B. A.; Clardy, J. Biflora-4,10(19),15-triene: a new diterpene from a termite soldier (Isoptera Termitidae Termitinae). *J. Org. Chem.* **1980**, ~~45(1)~~, 191-192~~;~~ DOI: 10.1002/chin.198025295.

77. Wiemer, D.F.; Meinwald, J.; Prestwich, G.D.; Miura, I. Cembrene A and (3Z) - cembrene A: diterpenes from a termite soldier (Isoptera Termitidae Termitinae). *J. Org. Chem.* **1980**, ~~44 (22)~~, 3950~~–~~3952~~;~~ DOI: 10.1002/chin.198013347.

78. Tempesta, M.S.; Pawlak, J.K.; Iwashita, T.; Naya, Y.; Nakanishi, K.; Prestwich, G.D. Cubugene, a diterpenoid with a novel carbon skeleton from a termite soldier (Isoptera Termitidae Termitinae). *J. Org. Chem.* **1984**, ~~49(11~~), 2077-2079~~;~~ DOI: 10.1002/chin.198452328.

79. Naya, Y.; Prestwich, G.D.; Spanton, S.G. Sesquiterpenes form termite soldiers. Structure of amiteol. A new 5 beta,7 beta,10 beta-eudesmane from *Amitermes excellens*. *Tetrahedron Lett.* **1982**, ~~23(30)~~, 3047-3050~~;~~ DOI: 10.1016/S0040-4039(00)87529-2.

80. Baker, R.; Coles, H.R.; Edwards, M.; Evans, D.A.; Howse, P.E.; Walmsley, S. Chemical composition of the frontal gland secretion of *Syntermes* soldiers (Isoptera, Termitidae). *J. Chem. Ecol.* **1981**, ~~7(1)~~, 135~~;~~ DOI: 10.1007/BF00988641.

81. Chuah, C.H. Interspecific variation in defense secretions of Malaysian termites from the genus *Bulbitermes*. *J. Chem. Ecol.* **2005**, ~~31(4)~~, 819~~-27;~~ DOI: 10.1007/s10886-005-3546-8.

82. Sillam-Dussès, D.; Sémon, E.; Robert, A.; Cancello, E.; Lenz, M.; Valterová, I.; Bordereau, C. Identification of multi-component trail pheromones in the most evolutionarily derived termites, the Nasutitermitinae (Termitidae). *Biol. J. Linn. Soc.* **2010**, ~~99(1)~~, 20-27~~;~~ DOI:

10.1111/j.1095-8312.2009. 01348.x.

83. Baker, R.; Organ, A.J.; Prout, K.; Jones, R. Isolation of a novel triacetoxysecotrinervitane from the termite *Constrictotermes cyphergaster* (Termitidae, sub-family Nasutitermitinae). *Tetrahedron Lett.* **1984**, ~~25(5)~~, 579-580~~;~~ DOI: 10.1016/S0040-4039(00)99943-X.

84. Lindström, M.; Norin, T.; Valterová, I.; Vrkoc, J. Chirality of the monoterpene alarm pheromones of termites. *Naturwissenschaften*. **1990**, ~~77(3)~~, 134-135~~;~~ DOI: 10.1007/BF01134477.

85. Baker, R.; Edwards, M.; Evans, D.A.; Walmsley, S. Soldier-specific chemicals of the termite *Curvitermes strictinasus* Mathews (Isoptera, Nasutitermitinae). *J. Chem. Ecol.* **1981**, ~~7(1)~~, 127~~-33;~~ DOI: 10.1007/BF00988640.

86. Canalis, E.; Lian, J.B. The monoterpenoid fraction of the defensive secretion in Nasutitermitinae from Papua New Guinea. *Biochem. Syst. Ecol.* **1988**, ~~16(4~~), 437-444~~;~~ DOI: 10.1016/0305-1978(88)90041-5.

87. ~~P.~~ Laurent; D. Daloze; ~~J. M.~~ Pasteels; ~~J. C.~~ Braekman. Trinervitene diterpenes from soldiers of two Nasutitermes species from French Guyana. *J. Nat. Prod.* **2005**, ~~68(4)~~, 532-536~~;~~ DOI: 10.1021/np040243v.

88. Everaerts, C.; Roisin, Y.; Bonnard, O.; Pasteels, J.M. Sesquiterpenes in the frontal gland secretions of nasute soldier termites from New Guinea. *J. Chem. Ecol.* **1993**, ~~19(12)~~, 2865-2879~~;~~ DOI: 10.1007/BF00980588.

89. Rabemanantsoa, A.; Ranarivelo, Y.; Andriantsiferana, M.; Tillequin, F.; Silverton, J.V.; Garraffo, H.M.; Spande, T.F.; Yeh, H.J.; Daly, J.W. A new secotrinervitane diterpene isolated from soldiers of the Madagascan termite species, *Nasutitermes canaliculatus*. *J. Nat. Prod.* **1996**, ~~59(9)~~, 883~~;~~ DOI: 10.1021/np960374z.

90. Valterová, I.; Vrkoč, J.; Norin, T. The enantiomeric composition of monoterpene hydrocarbons in the defensive secretions of Nasutitermes termites (Isoptera): Inter- and intraspecific variations. *Chemoecology* **1993**, ~~4(2)~~, 120-123~~;~~ DOI: 10.1007/BF01241682.

91. Chuah, C.H.; Goh, S.H.; Prestwich, G.D.; Tho, Y.P. Soldier defense secretions of the

Malaysian termite, *Hospitalioermes umbrinus* (Isoptera, Nasutitermitinae). *J. Chem. Ecol.* **1983**, ~~9(3)~~, 347-356~~;~~ DOI: 10.1007/BF00988454.

92. Chuah, C.H. Intraspecific variation in soldier defense secretions of *Longipeditermes longipes* (Isoptera, Nasutitermitinae). *Biochem. Syst. Ecol.* **2007**, ~~35(9)~~, 600-605~~;~~ DOI: 10.1016/j.bse.2007.04.003.

93. Honda, K. Osmeterial secretions of papilionid larvae in the genera *Luehdorfia*, *Graphium* and *Atrophaneura* (lepidoptera). *Insect Biochem.* **1980**, ~~10(5)~~, 583-588~~;~~ DOI: 10.1016/0020-1790(80)90095-5.

94. ~~H, Ô.~~; Noguchi, T.; Nehira, T. New oxygenated himachalenes in male-specific odor of the Chinese windmill butterfly, *Byasa alcinousalcinous*. *Nat. Prod. Res.* **2016**, ~~30(4)~~, 406~~;~~ DOI: 10.1080/14786419.2015.1019352.

95. Ômura, H.; Honda, K.; Feeny, P. From terpenoids to aliphatic acids: further evidence for late-instar switch in osmeterial defense as a characteristic trait of swallowtail butterflies in the Tribe Papilionini. *J. Chem. Ecol.* **2006**, ~~32(9)~~, 1999-2012~~;~~ DOI: 10.1007/s10886-006-9124-x.

96. Frankfater, C.; Tellez, M.R.; Slattery, M. The scent of alarm: ontogenetic and genetic variation in the osmeterial gland chemistry of *Papilio glaucus* (Papilionidae) caterpillars. *Chemoecology* **2009**, ~~19(2~~), 81-96~~;~~ DOI: 10.1007/s00049-009-0013-y.

97. Honda, K.; Hayashi, N. Chemical nature of larval osmeterial secretions of papilionid butterflies in the genera *Parnassius*, *Sericinus* and *Pachliopta*. *J. Chem. Ecol.* **1995**, ~~21(6)~~, 859~~-67;~~ DOI: 10.1007/BF02033466.

98. Honda, K. Evidence for *de novo* biosynthesis of osmeterial secretions in young larvae of the swallowtail butterflies (*Papilio*): deuterium incorporation *in vivo* into sesquiterpene hydrocarbons as revealed by mass spectrometry. *Int. J. Trop. Insect Sci.* **1983**, ~~4(3)~~, 255-261~~;~~ DOI: 10.1017/S1742758400001247.

99. Burger, B.V.; Munro, Z.; Spies, H.S.; Truter, V.; Geertsema, H.; Habich, A. Constituents of osmeterial secretion of pre-final instar larvae of citrus swallowtail, *Papilio demodocus*

(Esper) (Lepidoptera: Papilionidae). *J. Chem. Ecol.* **1985**, ~~11(8)~~, 1093-1113~~;~~ DOI: 10.1007/BF01020678.

100. Martínez, L.C.; Plata-Rueda, A.; Da, S.N.G.; Cossolin, J.F.; Dos Santos, M.H.; Zanuncio, J.C.; Serrão, J.E. Morphology, ultrastructure, and chemical compounds of the osmeterium of *Heraclides thoas* (Lepidoptera: Papilionidae). *Protoplasma*. **2018**, ~~6~~, 1-10~~;~~ DOI: 10.1007/s00709-018-1261-x.

101. Okumura, Y.; Ozeki, Y.; Itoh, T.; Ohta, S.; Ômura, H. Volatile terpenoids from male wings lacking scent scales in *Anthocharis scolymus* (Lepidoptera: Pieridae). *Appl. Entomol. Zool.* **2016**, ~~51(3)~~, 1-8~~;~~ DOI: 10.1007/s13355-016-0410-y.

102. Hayashi, N.; Kuwahara, Y.; Komae, H. The scent scale substances of male *Pieris* butterflies (*Pieris melete* and *Pieris napi*). *Experientia*. **1978**, ~~34(6)~~, 684-685~~;~~ DOI: 10.1007/BF01947255.

103. Kuwahara, Y. Scent scale substances of male *Pieris melete* Ménétriès (Pieridae: Lepidoptera). *Appl. Entomol. Zool.* **1979**, ~~14~~, 350-355~~;~~ DOI: 10.1303/aez.14.350.

104. Hayashi, N.; Nishi, A.; Murakami, T.; Maeshima, K.; Komae, H.; Sakao, T. The scent substances of *Pierid* butterflies (*Hebomoia glaucippe* Linnaeus) and the volatile components of their food plants (*Crataeva religiosa* Forst.). *Z. Naturforsch. C Bio. Sci.* **1985**, ~~40(1-2)~~, 47-50~~;~~ DOI:10.1515/znc-1985-1-211.

105. Burger, B.V.; Nell, A.E.; Smit, D.; Spies, H.S.; Mackenroth, W.M.; Groche, D.; Atkinson, P.R. Constituents of wing gland and abdominal hair pencil secretions of male African sugarcane borer, *Eldana saccharina* walker (Lepidoptera: Pyralidae). *J. Chem. Ecol.* **1993**, ~~19(10)~~, 2255-2277~~;~~ DOI: 10.1007/BF00979662.

106. Francke, W.; Schulz, S.; Sinnwell, V.; Koenig, W.A.; Roisin, Y. Epoxytetrahydroedulan, a new terpenoid from the hairpencils of Euploea (Lep.: Danainae) butterflies. *Eur. J. Org. Chem.* **1989**, ~~12~~, 1195-1201~~;~~ DOI: 10.1002/chin.199011288.

107. Schulz, S.; Nishida, R. The pheromone system of the male danaine butterfly, *Idea leuconoe*. *Bioorgan. Med. Chem.* **1996**, ~~4(3)~~, 341~~-9~~; DOI: 10.1007/BF02029947.

108. Dossey, A.T.; Walse, S.S.; Rocca, J.R.; Edison, A.S. Single-insect NMR: a new tool to probe chemical biodiversity. *ACS Chem. Biol.* **2006**, ~~1(8)~~, 511-514~~;~~ DOI: 10.1021/cb600318u.

109. Happ, G.M.; Strandberg, J.D.; Happ, C.M. The terpene-producing glands of a Phasmid insect. Cell morphology and histochemistry. *J. Morphol.* **1966**, ~~119(2)~~, 143-160~~;~~ DOI: 10.1002/jmor.1051190204.

110. Prescott, T.A.K.; Bramham, J.; Zompro, O.; Maciver, S.K. Actinidine and glucose from the defensive secretion of the stick insect *Megacrania nigrosulfurea*. *Biochem. Syst. Ecol.* **2009**, ~~37(6)~~, 759-760~~;~~ DOI: 10.1016/j.bse.2009.11.002.

111. Bouchard, P.; Hsiung, C.C.; Yaylayan, V.A. Chemical analysis of defense secretions of *Sipyloidea sipylus* and their potential use as repellents against rats. *J. Chem. Ecol.* **1997**, ~~23(8)~~, 2049-2057~~;~~ DOI: 10.1023/B:JOEC.0000006488.58081.66.

112. Agosti, D.; Austin, C.; Gökçen, O.A.; König, W.A.; Morgan, E.D.; Scott, E.D.; Wehner, R. (S)-2-Methyl-1-hexanol, characteristic mandibular gland substance of ants of the *Cataglyphis bicolor* group. *Chemoecology*. **1996**, ~~7(1)~~, 57-60~~;~~ DOI: 10.1007/BF01240638.

113. Hayashi, N.; Komae, H. Components of the ant secretions. *Biochem. Syst. Ecol.* **1980**, ~~8(3)~~, 293-295~~;~~ DOI: 10.1016/0305-1978(80)90063-0.

114. Bernardi, R.; Cardani, C.; Ghiringhelli, D.; Selva, A.; Baggini, A.; Pavan, M. On the components of secretion of mandibular glands of the ant *Lasius* (Dendrolasius) *fuliginosus*. *Tetrahedron Lett.* **1967**, ~~8(40)~~, 3893-3896~~;~~ DOI: /10.1016/S0040-4039(01)89747-1.

115. Brand, J.M.; Blum, M.S.; Lloyd, H.A.; Fletcher, D.J.C. Monoterpene hydrocarbons in the poison gland secretion of the ant *Myrmicaria natalensis* (Hymenoptera: Formicidae). *Ann. Entomol. Soc. Am.* **1974**, ~~67(3)~~, 525-526~~;~~ DOI: 10.1093/aesa/67.3.525.

116. Keegans, S.J.; Billen, J.; Morgan, E.D.; Gökcen, O.A. Volatile glandular secretions of three species of new world army ants, *Eciton burchelli*, *Labidus coecus*, and *Labidus praedator*. *J. Chem. Ecol.* **1993**, ~~19(11)~~, 2705-~~19;~~ DOI: 10.1007/BF00980702.

117. Bergström, G.; Löfqvist, J. Chemical basis for odour communication in four species of

Lasius ants. *J. Insect Physiol.* **1970**, 16(12), 2353-2375; DOI: 10.1016/0022-1910(70)90157-5.

118. Bergström, G.; Löfqvist, J. Chemical congruence of the complex odoriferous secretions from Dufour's gland in three species of ants of the genus *Formica*. *J. Insect Physiol*. **1973**, 19(4), 877-907; DOI: 10.1016/0022-1910(73)90159-5.

119. Edwards, J.P.; Chambers, J. Identification and source of a queen-specific chemical in the pharaoh's ant, *Monomorium pharaonis* (L.). *J. Chem. Ecol.* **1984**, 10(12), 1731-1747; DOI: 10.1007/BF00987358.
